# Autophagy is required for the development and functionality of lacrimal gland-like organoids

**DOI:** 10.1016/j.stemcr.2025.102744

**Published:** 2025-12-18

**Authors:** Gamze Kocak, Miriam E. Korsgen, Leticia F. Amores, Congxin Sun, Merve Ceylan, Asmaa Ghazwani, Merve Kandirici, Malgorzata Zatyka, Elena Seranova, Animesh Acharjee, Timothy Barrett, Bayram Yuksel, Adil Mardinoglu, Sinan Güven, Sovan Sarkar

**Affiliations:** 1 Department of Cancer and Genomic Sciences, School of Medical Sciences, College of Medicine and Health, University of Birmingham, Birmingham, UK; 2 Izmir International Biomedicine and Genome Institute, Dokuz Eylül University, Izmir, Türkiye; 3 Izmir Biomedicine and Genome Center, Izmir, Türkiye; 4 Genetic Diagnosis Center, SZAOMICS, Istanbul, Türkiye; 5 NMN Bio Ltd., Walker House, Liverpool, UK; 6 Centre for Health Data Research, University of Birmingham, Birmingham, UK; 7 Institute of Translational Medicine, University Hospitals Birmingham NHS Foundation Trust, Birmingham, UK; 8 Department of Endocrinology, Birmingham Women’s and Children’s Hospital, Steelhouse Lane, Birmingham, UK; 9 Science for Life Laboratory, KTH–Royal Institute of Technology, Stockholm, Sweden; 10 Centre for Host-Microbiome Interactions, Faculty of Dentistry, Oral & Craniofacial Sciences, King’s College London, London, UK; 11 Department of Medical Biology and Genetics, Faculty of Medicine, Dokuz Eylül University, Izmir, Türkiye

**Keywords:** autophagy, cell death, differentiation, development, hESC, organoid, SEAM, lacrimal gland, PAX6, NMN

## Abstract

Lacrimal glands (LGs) serve as pivotal exocrine glands crucial for protecting the ocular surface. Dysfunction in LG cell composition or secretion is implicated in dry eye disease (DED). While autophagy plays a vital role in tissue homeostasis in many organs, how it affects LG development and secretory function is not known. Here, we have undertaken a genetic study by utilizing autophagy-deficient human embryonic stem cells (hESCs) and differentiating them into LG-like organoids. Autophagy-deficient LG-like organoids exhibited improper development and secretion, along with increased protein aggregation, proliferation, and cell death. These phenotypes were associated with an accumulation of PAX6, a transcription factor crucial for brain and eye development, which we identified as an autophagy substrate. Pharmacological interventions with nicotinamide mononucleotide (NMN) and melatonin were able to rescue the cellular dysfunction in autophagy-deficient LG-like organoids. Together, our study highlights the role of autophagy in LG along with potential therapeutic interventions for DED.

## Highlights

• Loss of autophagy impairs development and secretion of *ATG5*^−/−^ LG organoids
• *ATG5*^−/−^ LG organoids exhibit elevated cell death, proliferation, and aggresomes
• PAX6, identified as an autophagy substrate, accumulates in *ATG5*^−/−^ LG organoids
• Pharmacological interventions rescue cellular dysfunction in *ATG5*^−/−^ LG organoids

## Graphical abstract

**Figure:**
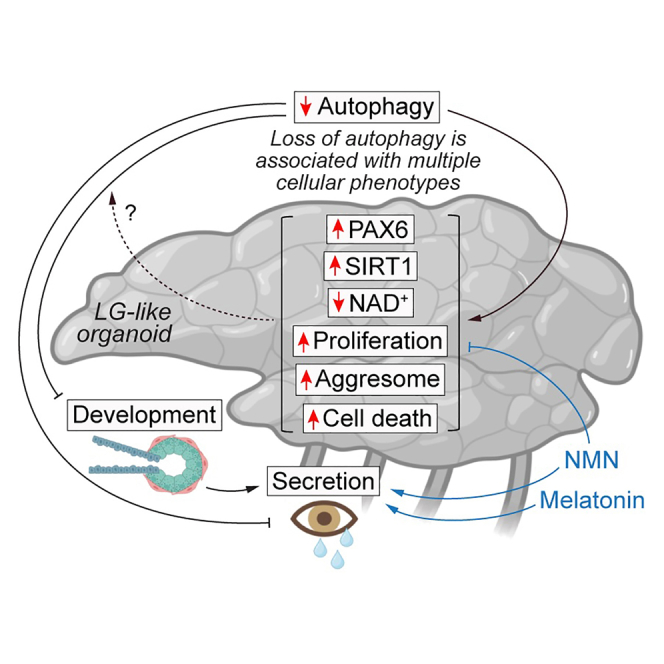

## Teaser

Kocak et al. generated lacrimal gland (LG)-like organoids with autophagy deficiency that exhibited improper development and secretory function. This was associated with increased PAX6, protein aggregation, proliferation, and cell death. These cellular dysfunctions could be rescued pharmacologically, thus providing insights for therapeutic interventions in dry eye disease.

## Introduction

The lacrimal gland (LG) is an exocrine gland responsible for producing the aqueous component of tear film, which is essential for maintaining ocular surface moisture and homeostasis. Proper functioning of LG relies on the coordinated organization of acinar, ductal, and myoepithelial cells (Zoukhri, 2010). Dysfunction in LG cell composition or secretion is implicated in dry eye disease (DED), in which current treatment options offer only short-term relief (Messmer, 2015).

Advancements in tissue engineering and regenerative medicine have facilitated the study of LG physiology and functionality through the culture of embryonic and adult cells. Notably, LGs exhibit regenerative capabilities, harboring stem/progenitor cells within the tissue (Bannier-Hélaouët et al., 2021). Challenges in isolating the regenerative cells from LG tissue underscore the importance of utilizing human pluripotent stem cells (hPSCs) for research on regenerative therapy and drug discovery pertaining to LGs. The generation of LG organoid models from hPSCs is particularly promising for providing insights into the developmental processes and molecular mechanisms (Asal et al., 2023; Hayashi et al., 2022). Organoid models generated from hPSCs provide a physiologically relevant human *in vitro* model to reliably recapitulate structural and functional aspects of LG for studying physiology, molecular pathways, and pharmacological interventions (Clevers, 2016).

One of the biological processes that is critical for gland tissue development and functionality is autophagy, an intracellular catabolic process essential for the maintenance of cellular and energy homeostasis (Aman et al., 2021; Morgan-Bathke et al., 2015). Autophagy involves the formation of autophagosomes that sequester undesirable macromolecules and organelles, followed by their fusion with the lysosomes for degradation of autophagic cargo and recycling of breakdown products. This multi-step process is orchestrated by several autophagy-related proteins (ATGs) that form distinct functional complexes: ULK1 kinase complex regulating autophagy initiation, class III PI3K complex facilitating phagophore nucleation, ATG12-ATG5-ATG16L1 conjugation system driving membrane expansion, and LC3/ATG8 lipidation system enabling autophagosome formation and cargo recognition (Aman et al., 2021). During tissue formation and morphogenesis, autophagy contributes to cellular and tissue homeostasis by eliminating damaged proteins and organelles. Conversely, tissue-specific abrogation of autophagy in mice by knockout of essential autophagy genes (*Atg5* or *Atg7*) results in dysfunction or degeneration of the affected organs (Mizushima and Levine, 2010). Given these fundamental roles of autophagy, emerging evidence suggests a potential involvement of autophagy in LG function. For instance, a tear glycoprotein called lacritin has been implicated in autophagy induction during inflammatory stress (Wang et al., 2013), whereas autophagy activation has been associated with acinar cell protection and LG size maintenance in DED (Seo et al., 2014). However, the precise role of autophagy in LG development and function remains elusive.

As an essential component of the autophagic machinery required for autophagosome formation, knockout of the *ATG5* gene provides a robust and well-established approach to genetically disrupt autophagy (Kuma et al., 2017). Here, we investigated the impact of loss of autophagy on LG development and its secretory function using autophagy-deficient (*ATG5*^−/−^) LG-like organoids, which were differentiated from genome-edited *ATG5*^−/−^ human embryonic stem cells (hESCs). We also examined the therapeutic potential of pharmacological agents in improving the cellular phenotypes and secretory function of LG-like organoids with autophagy deficiency.

## Results

### Autophagy-deficient hESCs exhibit improper LG differentiation

**Figure 1:**
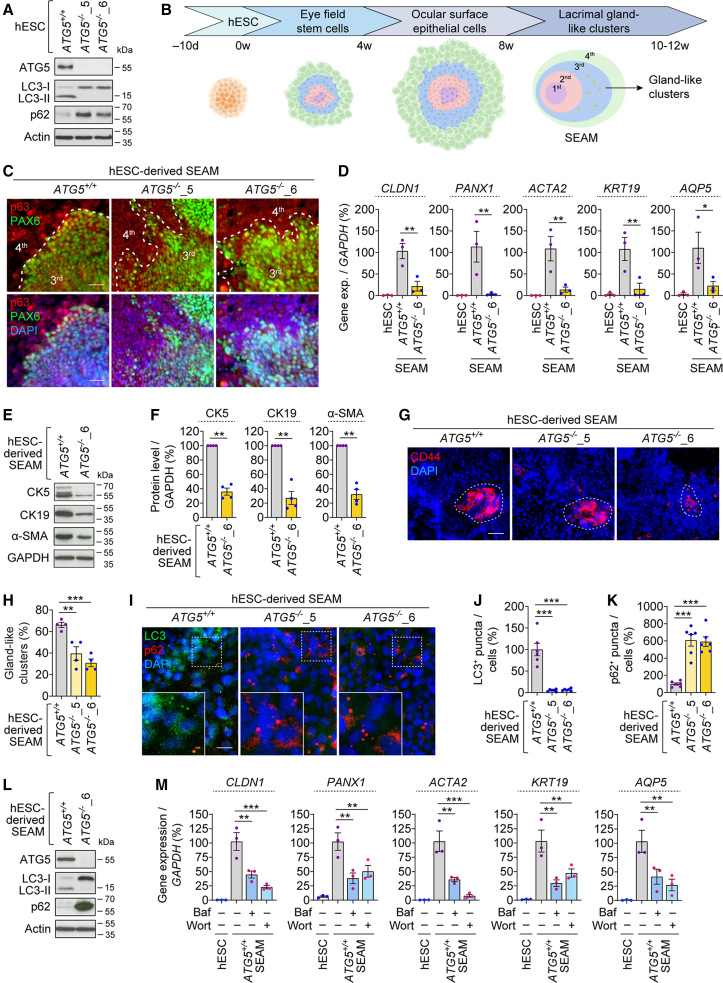
Autophagy-deficient hESCs exhibit improper LG differentiation and SEAM formation (A) Immunoblotting analyses of ATG5, LC3, and p62 in *ATG5*^+/+^, *ATG5*^−/−^_5, and *ATG5*^−/−^_6 hESCs. (B) Schematic illustration of SEAM formation from hESCs. (C–L) Immunofluorescence images of PAX6 and p63 (C); gene expression analyses of *CLDN1*, *PANX1*, *ACTA2*, *KRT19*, and *AQP5* relative to *GAPDH* (D); immunofluorescence images of CD44 (E) and quantification of CD44^+^ gland-like clusters (F); and immunofluorescence images of LC3 and p62 (G) in *ATG5*^+/+^, *ATG5*^−/−^_5, and *ATG5*^−/−^_6 hESC-derived SEAM as indicated. Wild-type hESCs were used as negative control (D). (M) Gene expression analysis of *CLDN1*, *PANX1*, *ACTA2*, *KRT19*, and *AQP5* relative to *GAPDH* in *ATG5*^*+/+*^ hESC-derived SEAM, treated with DMSO (vehicle control), 50 nm bafilomycin A_1_ (Baf), or 1 μM wortmannin (Wort) for the last 10 days of the 8-week differentiation period. Wild-type hESCs used as negative control. Graphical data are mean ± SEM of *n* = 3–6 experimental replicates from 3 independent experiments; *p* values calculated by unpaired two-tailed Student’s *t* test (F) or one-way ANOVA followed by multiple comparisons with the two-stage linear step-up procedure of Benjamini, Krieger, and Yekutieli (D, H, J, K, and M). ^∗^*p* < 0.05; ^∗∗^*p* < 0.01; ^∗∗∗^*p* < 0.001. Scale bar: 50 μm (C and I) and 100 μm (G). See also Figure S1.

To study the role of autophagy on LG development and its secretory function, we utilized wild-type (*ATG5*^+/+^) and autophagy-deficient (*ATG5*^−/−^) hESCs, which we previously generated by knockout of *ATG5* exon 3 via genome editing (Sun et al., 2023). Loss of ATG5 prevents autophagosome formation (as seen by autophagosome marker LC3-II) and retards autophagic flux, leading to accumulation of autophagy substrates (such as p62; Figure S1A). Loss of autophagy was confirmed in two independent clones of *ATG5*^−/−^ hESCs (clones #5 and #6) under basal condition by the absence of ATG5 and LC3-II and accumulation of p62 (Figures 1A and S1B). The pluripotency of *ATG5*^+/+^ and *ATG5*^−/−^ hESCs was confirmed by immunofluorescence and gene expression analyses of pluripotency markers: OCT4, SOX2, and NANOG (Figures S1C and S1D). The proliferative capacity of *ATG5*^−/−^ hESCs, analyzed by proliferation marker Ki-67, was comparable to wild-type hESCs (Figures S1E and S1F).

Multiple studies have shown that LG-like organoids can be generated from hPSCs using multi-zonal ocular organoid differentiation, which is one of the strategies to mimic whole-eye development (Asal et al., 2023; Hayashi et al., 2022; Li et al., 2019). We generated self-formed, ectodermal, autonomous, multi-zone (SEAM) organoids that contain precursor cells of a range of ocular tissue lineages, using the differentiation protocol adapted from previous studies (Hayashi et al., 2022) (Figures 1B and S1G). At 6-week differentiation, clusters of cells emerged in SEAM zone 3 that expressed ocular surface epithelial-like cell markers PAX6 and p63, along with zone 4 that expressed only p63 (Figure 1C) (Hayashi et al., 2022), suggesting the generation of multi-zonal cells in both *ATG5*^+/+^ and *ATG5*^−/−^ hESC-derived SEAM. However, zones 3 and 4 were less organized in *ATG5*^−/−^ SEAM, displaying increased PAX6 levels, while p63 immunostaining remained comparable to wild type (Figures 1C and S1H).

We next studied the expression of LG-related markers in hESC-derived SEAM to investigate the effects of loss of autophagy on LG progenitors. LG is composed of three major cell types: acinar, ductal, and myoepithelial cells. These cell types express cytokeratin 5 (CK5; LG progenitor marker), while luminal ductal cells express cytokeratin 19 (CK19), and myoepithelial cells secrete α-smooth muscle actin (α-SMA) (Basova et al., 2020). The protein levels of CK5, CK19, and α-SMA, along with the gene expression of LG-related acinar/ductal (*CLDN1*, *AQP5*, *PANX1*, and *KRT19* encoding CK19) and myoepithelial (*ACTA2* encoding α-SMA) cell markers, were decreased in *ATG5*^−/−^ SEAM compared to *ATG5*^*+/+*^ SEAM (Figures 1D–1F). Moreover, gland-like clusters were less in *ATG5*^−/−^ SEAM, as analyzed by the gland cell marker CD44 (Figures 1G and 1H). To assess the secretory capacity of these gland-like structures, we measured peroxidase enzyme that was found to be diminished in *ATG5*^−/−^ SEAM (Figure S1I). We confirmed autophagy deficiency in *ATG5*^−/−^ SEAM via lack of ATG5 and LC3-II, as well as accumulation of p62 that did not show any changes in its gene expression (*SQSTM1*; Figures 1I–1L, S1J, and S1K). These data raise the possibility of improper LG differentiation in autophagy-deficient SEAM.

The phenotypes arising from genetic abrogation of autophagy were confirmed by chemical disruption of this process at distinct stages, such as with the autophagy blocker bafilomycin A_1_ (prevents autophagosome-lysosome fusion and lysosomal acidification) and the autophagy inhibitor wortmannin (inhibits autophagosome biogenesis) (Panda et al., 2019). Consistent with the *ATG5*^−/−^ SEAM phenotype (Figure 1D), gene expression of acinar/ductal and myoepithelial cell markers was also decreased following treatment with bafilomycin A_1_ and wortmannin in *ATG5*^*+/+*^ SEAM (Figure 1M). This suggests that these effects are due to inhibition of autophagy and not because of autophagy-independent roles of ATG5.

### Perturbations in gene expression in autophagy-deficient hESC-derived SEAM

**Figure 2:**
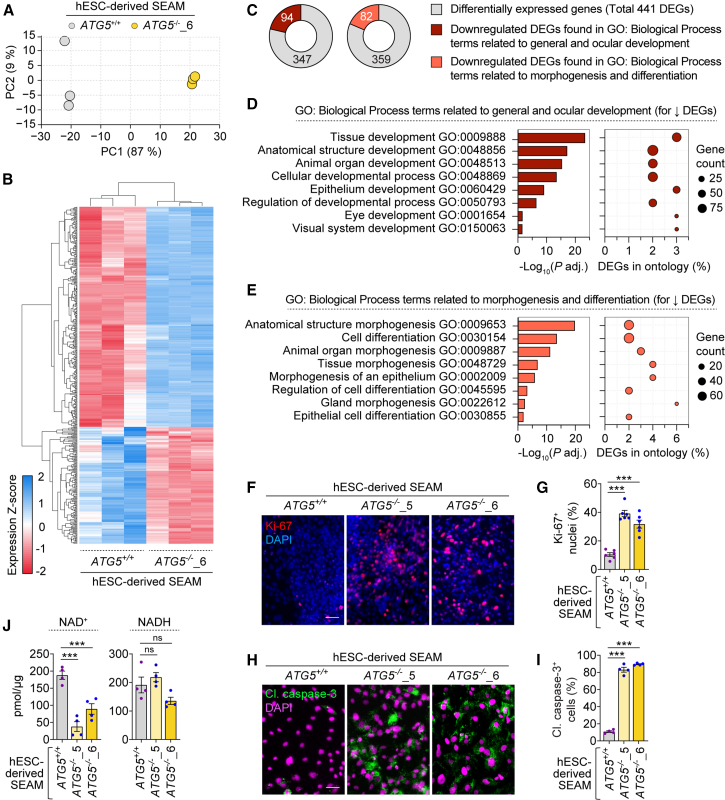
Autophagy-deficient hESC-derived SEAM exhibits altered gene expression and various cellular phenotypes (A and B) PCA scores plot of gene expression (A), and heatmap with hierarchical clustering of RNA-seq expression Z-scores for differentially expressed genes (DEGs) (B) in *ATG5*^+/+^ and *ATG5*^−/−^_6 hESC-derived SEAM. (C–E) Pie charts (C) and bar and dot plots (D and E) of downregulated DEGs enriched in GO: Biological Process terms related to general and ocular development (C and D) or morphogenesis and differentiation (C and E) in *ATG5*^−/−^_6 hESC-derived SEAM compared to *ATG5*^+/+^ hESC-derived SEAM. The total number of downregulated DEGs in selected GO: Biological Process terms compared to all 441 DEGs is indicated (C). (F–J) Immunofluorescence images of Ki-67 (F) and cleaved caspase-3 (H); quantification of Ki-67^+^ nuclei (G) and cleaved caspase-3^+^ cells (I); and measurement of NAD^+^ and NADH levels (J) in *ATG5*^+/+^, *ATG5*^−/−^_5, and *ATG5*^−/−^_6 hESC-derived SEAM. Graphical data are mean ± SEM of *n* = 4–6 experimental replicates from 3 independent experiments; *p* values calculated by one-way ANOVA followed by multiple comparisons with the two-stage linear step-up procedure of Benjamini, Krieger, and Yekutieli (G, I, and J). For transcriptomics analysis (*n* = 3 experimental replicates from 3 independent experiments), the threshold for differential gene expression was set, considering the Benjamini-Hochberg *p* adj. value < 0.05, |Log2(foldchange)| > 1 as significant (B and C). GO: Biological Process terms were selected based on corrected *p* value < 0.05, using the g:SCS multiple testing correction method. The dot sizes indicate the number of DEGs (D and E). ^∗∗∗^*p* < 0.001; ns, non-significant. Scale bar: 100 μm (F and H). See also Figure S2.

To gain insights on whether the developmental and differentiation processes are perturbed by autophagy deficiency, we conducted transcriptional analysis using bulk RNA-seq on *ATG5*^+/+^ and *ATG5*^−/−^ hESC-derived SEAM. Principal component analysis (PCA) and hierarchical clustering heatmap revealed distinct group patterns (Figures 2A and 2B), indicating apparent transcriptional variation between *ATG5*^+/+^ and *ATG5*^−/−^ SEAM. Despite an outlier in the wild-type group in the PCA plot (Figure 2A), which is possibly due to the multicellular nature and inherent heterogeneity of SEAM, the heatmap demonstrates clear clustering of *ATG5*^+/+^ and *ATG5*^−/−^ groups (Figure 2B). Gene Ontology (GO) analysis was performed on 441 differentially regulated genes (DEGs) to identify the biological processes affected. Specifically, we focused on downregulated DEGs associated with development, morphogenesis, and differentiation processes. We found 94 DEGs related to general and ocular development processes and 82 DEGs involved in morphogenesis and differentiation processes that were significantly downregulated in *ATG5*^−/−^ SEAM (Figures 2C–2E). These transcriptomics data support our findings that LG differentiation is likely impaired in autophagy-deficient SEAM.

We previously showed that loss of autophagy in hESCs and hESC-derived neurons mediates cytotoxicity via depletion of total nicotinamide adenine dinucleotide (NAD) pool (Sun et al., 2023). Therefore, we analyzed the transcriptomics dataset for perturbations in processes related to cell death, NAD metabolism, as well as cellular proliferation. GO analysis revealed 73 downregulated and 52 upregulated DEGs related to cell death and proliferation and 14 upregulated DEGs related to NAD metabolism in *ATG5*^−/−^ SEAM (Figures S2A–S2F), suggesting that these biological processes are affected. Downregulation of DEGs associated with negative regulation of cell death and proliferation and upregulation of DEGs related to apoptotic pathway and proliferation are suggestive of increased cell death and proliferation in *ATG5*^−/−^ SEAM (Figures S2B and S2C). We respectively confirmed these phenotypes in *ATG5*^−/−^ SEAM that showed elevation in cleaved caspase-3 and Ki-67 (Figures S2F–S2I), along with depletion of NAD^+^ levels (Figure 2J). It is plausible that alterations in cell viability linked to NAD^+^ levels, along with cellular proliferation, might impact the multi-zonal ocular cells differentiation for LG formation.

### Autophagy deficiency impairs hESC-derived LG-like organoid development and secretory function

**Figure 3:**
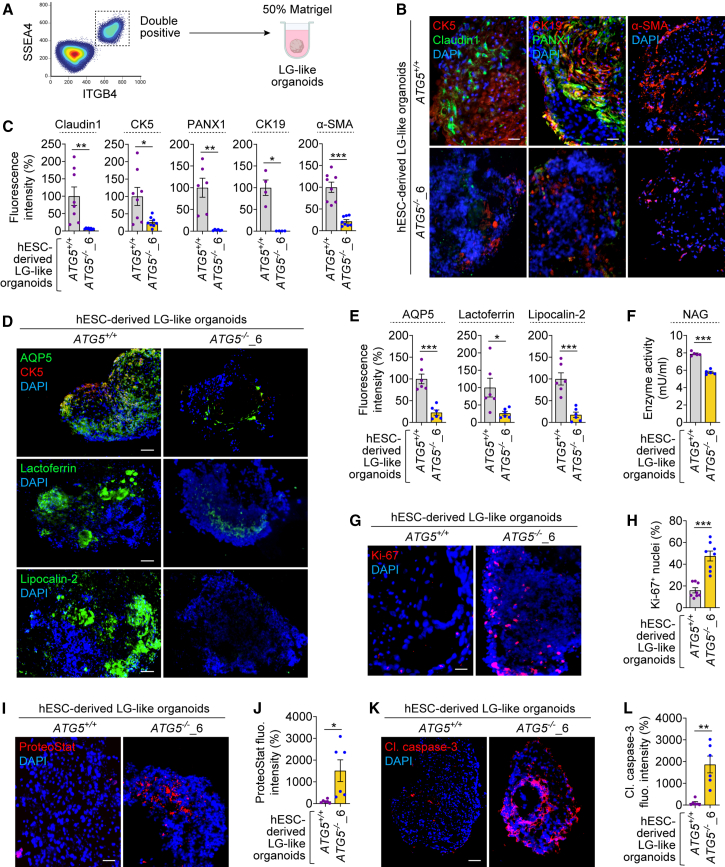
Loss of autophagy perturbs cellular and functional phenotypes of hESC-derived LG-like organoids (A) Schematic illustration of LG-like organoid generation from SEAM. (B–L) Immunofluorescence images of CK5, Claudin1, PANX1, CK19, and α-SMA (B) and AQP5, CK5, lactoferrin, and lipocalin-2 (D) and quantification of fluorescence intensity (C and E); NAG enzyme activity (F); immunofluorescence images of Ki-67 (G) and quantification of Ki-67^+^ cells (H); fluorescence images of ProteoStat staining (I) and quantification of fluorescence intensity (J); and immunofluorescence images of cleaved caspase-3 (K) and quantification of fluorescence intensity (L) in *ATG5*^+/+^ and *ATG5*^−/−^_6 hESC-derived LG-like organoids. Graphical data are mean ± SEM of *n* = 5–8 experimental replicates from 3 independent experiments; *p* values calculated by unpaired two-tailed Student’s *t* test (C, E, F, H, J, and L). ^∗^*p* < 0.05; ^∗∗^*p* < 0.01; ^∗∗∗^*p* < 0.001. Scale bar: 50 μm (B, G, and I) and 100 μm (D and K). See also Figure S3.

We generated LG-like organoids from *ATG5*^+/+^ and *ATG5*^−/−^ hESC-derived SEAM to investigate the role of autophagy in LG development and functionality using a three-dimensional (3D) culture. We first obtained SSEA4/ITGB4 double-positive cells using flow cytometry from hESC-derived SEAM after 10–12 weeks of differentiation (Figures 3A, S3A, and S3B), which identifies ocular surface epithelial stem cells (Hayashi et al., 2022). The cells were then processed for 3D culture after spheroid formation for 1 day, differentiated for 1 month, and embedded in Matrigel to generate LG-like organoids (Figure 3A). Immunostaining revealed that *ATG5*^−/−^ hESC-derived LG-like organoids showed reduction in the expression of acinar/ductal (Claudin1, CK5, PANX1, and CK19) and myoepithelial (α-SMA) cell markers (Figures 3B and 3C), suggesting improper formation of these cell types. Additionally, we analyzed hESC-derived SEAM and LG-like organoid formation in another *ATG5*^−/−^ hESC clonal line (clone #5), which exhibited similar reduction in LG-related markers by gene expression and immunofluorescence analyses (Figures S3C–S3E). These data imply that autophagy is required for LG development.

We next examined the impact of autophagy deficiency on the secretory function of hESC-derived LG-like organoids. LG is responsible for the secretion of the tear proteins like lactoferrin, lipocalin-2, and lysozyme, as well as water by the water channel protein aquaporin-5 (AQP5) expressed by LG acinar cells (Bron et al., 2017). We found that the expression of lactoferrin, lipocalin-2, and AQP5 was decreased in *ATG5*^−/−^ LG-like organoids compared to *ATG5*^*+/+*^ organoids (Figures 3D and 3E). This suggests that loss of autophagy impairs the expression of LG secretory proteins, implicating that their secretory function could be affected. To further analyze the secretory function, we measured the activity of N-acetyl-β-*d*-glucosaminidase (NAG) enzyme, which is present during lysozyme secretion in tears (Asal et al., 2023). Using β-hexosaminidase assay, we found that *ATG5*^−/−^ LG-like organoids had lower NAG enzyme activity compared to *ATG5*^*+/+*^ organoids (Figure 3F), suggesting impairment in lysozyme secretion due to lack of autophagy.

We further studied additional cellular phenotypes. As in SEAM, *ATG5*^−/−^ LG-like organoids showed elevation in Ki-67^+^ cells (Figures 3G and 3H), suggesting increase in cellular proliferation. We next analyzed proteostasis by analyzing aggresomes (accumulation of aggregated proteins) in LG-like organoids using ProteoStat staining. Since autophagy degrades aggregated proteins (a process called aggrephagy), dysfunction in this degradative process can lead to proteotoxic stress (Goldberg, 2003). We previously demonstrated profound build-up of aggresomes due to improper aggrephagy in autophagy-deficient hESCs and hESC-derived neurons (Sun et al., 2023). Likewise, *ATG5*^−/−^ LG-like organoids displayed elevation in ProteoStat signals (Figures 3I and 3J), suggesting accumulation of aggresomes that is expected due to lack of autophagy. Finally, we assessed cell viability by immunostaining for cleaved caspase-3 and TUNEL staining for apoptotic nuclei. *ATG5*^−/−^ LG-like organoids showed higher cleaved caspase-3 signals and TUNEL^+^ apoptotic nuclei compared to *ATG5*^*+/+*^ organoids (Figures 3K, 3L, S3F, and S3G), suggesting increased cytotoxicity associated with loss of autophagy. Collectively, our data suggest that autophagy-deficient LG-like organoids are associated with multiple cellular phenotypes that could affect their formation.

### PAX6 is an autophagy substrate that accumulates in autophagy-deficient SEAM and LG-like organoids

**Figure 4:**
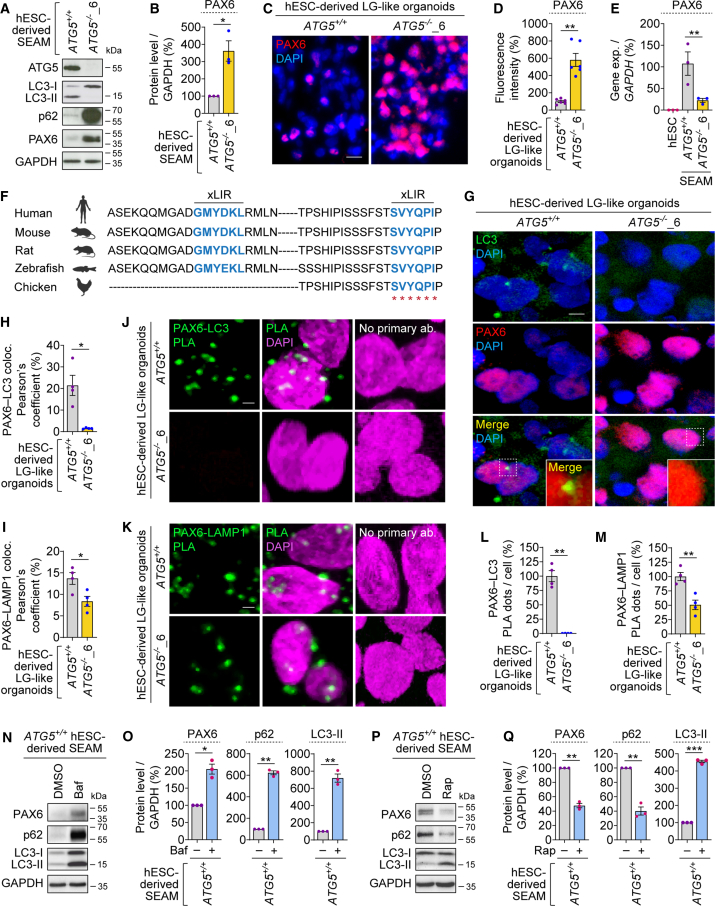
PAX6 is an autophagy substrate that accumulates in autophagy-deficient hESC-derived SEAM and LG-like organoids (A–E) Immunoblotting analyses of ATG5, p62, and PAX6 (A); densitometric analysis of PAX6 relative to GAPDH (B); immunofluorescence images of PAX6 (C) and quantification of fluorescence intensity (D); and gene expression analysis of *PAX6* relative to *GAPDH* (E) in *ATG5*^+/+^ and *ATG5*^−/−^_6 hESC-derived SEAM (A, B, and E) or LG-like organoids (C and D). Wild-type hESCs used as negative control (E). (F) Potential LIR motifs from the iLIR database and multiple sequence alignment from species. Residues conserved in all five species shown are highlighted by asterisks (^∗^). (G–M) Immunofluorescence images of PAX6 and LC3 (G), Pearson’s coefficient of PAX6-LC3 (H) and PAX6-LAMP1 (I) colocalization, and proximity ligation assay (PLA) fluorescent signal images (J and K) and quantification of PLA dots per cell (L and M) of PAX6-LC3 and PAX6-LAMP1 PLA in *ATG5*^+/+^ and *ATG5*^−/−^_6 hESC-derived LG-like organoids. (N–Q) Immunoblotting (N and P) and densitometric (O and Q) analyses of PAX6, p62, and LC3 relative to GAPDH in *ATG5*^*+/+*^ hESC-derived SEAM, treated with DMSO (vehicle control), 400 nM bafilomycin A_1_ (Baf), or 1 μM rapamycin (Rap) for 72 h, as indicated. Different exposures are shown for PAX6 and p62 to visualize differences (N and P). Graphical data are mean ± SEM of *n* = 3–6 experimental replicates from 3 independent experiments; *p* values calculated by unpaired two-tailed Student’s *t* test (B, D, H, I, L, M, O, and Q) or one-way ANOVA followed by multiple comparisons with the two-stage linear step-up procedure of Benjamini, Krieger, and Yekutieli (E). ^∗^*p* < 0.05, ^∗∗^*p* < 0.01. Scale bar: 10 μm (C, G, J, and K). See also Figure S4.

PAX6 is a transcription factor that is crucial for brain and eye development (Georgala et al., 2011; Kozmik, 2005). Genetic studies in mice have shown that Pax6 is indispensable for LG development and tear production (Bannier-Hélaouët et al., 2021). During LG differentiation, PAX6 is expressed in the SEAM structure (Figures 1C and S1G) and in LG. While multiple LG-related markers in *ATG5*^−/−^ hESC-derived SEAM and LG-like organoids were markedly downregulated at gene and protein levels (Figures 1D–1F, 3B, 3C, and S3C–S3E), we surprisingly found profound accumulation of PAX6 protein (Figures 1C, 4A–4D, and S1H). However, *PAX6* gene expression was decreased in *ATG5*^−/−^ SEAM (Figure 4E). This unexpected finding suggests that accumulation of PAX6 in autophagy-deficient cells, which also exhibited buildup of the autophagy substrate p62 (Figure 4A), occurs at the protein level, likely due to its impaired degradation.

Since PAX6 accumulates upon loss of autophagy, we hypothesized that this protein might be an autophagy substrate. During the process of selective autophagy, specific autophagic cargo is captured within the autophagosomes by interacting with the LC3 family member proteins via a unique sequence motif called LC3-interaction region (xLIR) (Birgisdottir et al., 2013). To investigate whether PAX6 has putative LIR motifs, we searched the iLIR database, which is a web resource for LIR motif-containing proteins in eukaryotes (Jacomin et al., 2016). Based on the *in silico* analysis, we found that PAX6 has two LIR motifs that are evolutionarily conserved among various species (Figure 4F). To assess if PAX6 colocalizes with autophagosomes and is subsequently delivered to the lysosomes, we performed Pearson’s correlation coefficient between PAX6 and the autophagosome marker LC3 or the lysosomal marker LAMP1 by co-immunostaining. We found that PAX6 colocalized with LC3 in wild-type LG-like organoids but not in *ATG5*^−/−^ organoids due to lack of autophagosomes (Figures 4G, 4H, and S4A). This colocalization occurred in proximity to the nucleus (Figures 4G and S4A), raising the possibility that nuclear PAX6 undergoes nucleophagy, a selective autophagy process for removal of nuclear material (Papandreou and Tavernarakis, 2019). PAX6 also colocalized with LAMP1 in both *ATG5*^+/+^ and *ATG5*^−/−^ LG-like organoids, although the colocalization was reduced in *ATG5*^−/−^ organoids, probably due to fewer lysosomes (Figures 4I and S4B). To further validate PAX6-LC3 and PAX6-LAMP1 associations, we performed proximity ligation assay (PLA). Wild-type LG-like organoids exhibited distinct PAX6-LC3 and PAX6-LAMP1 PLA signals (Figures 4J–4M), suggesting that PAX6 associates with autophagosomes and lysosomes. However, *ATG5*^−/−^ LG-like organoids had no PAX6-LC3 but fewer PAX6-LAMP1 PLA signals (Figures 4J–4M), similar to the observations from co-immunostaining studies (Figures 4J–4I and S4B). These data suggest that PAX6 could be a selective autophagy substrate that may undergo nucleophagy and subsequent lysosomal degradation.

To ascertain autophagic clearance of PAX6, we assessed autophagy modulators such as bafilomycin A_1_ (autophagy blocker; retards autophagosome maturation) and rapamycin (autophagy inducer; stimulates autophagosome biogenesis) (Panda et al., 2019). Preventing autophagic flux with bafilomycin A_1_ in wild-type SEAM increased PAX6 levels (Figures 4N and 4O), thereby recapitulating the *ATG5*^−/−^ SEAM phenotype (Figures 4A and 4B), along with accumulation of p62 and LC3-II as expected (Figures 4N and 4O). Conversely, enhancing autophagic flux with rapamycin in wild-type SEAM decreased PAX6 and p62 levels, along with an increase in LC3-II levels due to more autophagosome formation (Figures 4P and 4Q). These findings implicate that PAX6 undergoes autophagic degradation, and its accumulation in *ATG5*^−/−^ LG-like organoids correlates with improper LG differentiation.

We further studied whether abrogation of autophagy affects PAX6 function by analyzing the expression of its target genes: *SOX2*, *FOXC1*, *TGFB2*, and *BMP4* (Coutinho et al., 2011). In *ATG5*^−/−^ SEAM, the gene expression of *SOX2*, *FOXC1*, and *TGFB2* was upregulated, while that of *BMP4* was reduced (Figure S4C), as expected due to PAX6 buildup (Figures 4A and 4B). This implies that PAX6 accumulation during autophagy deficiency leads to a functional imbalance in its signaling that may affect LG differentiation.

### Pharmacological rescue of cellular and functional phenotypes in autophagy-deficient LG-like organoids

We investigated whether the cellular and functional phenotypes in autophagy-deficient LG-like organoids could be rescued by pharmacological interventions. Due to loss of autophagy in *ATG5*^−/−^ organoids, we could not use autophagy inducers to study the rescue effects because these agents will not have any autophagy-dependent impact. Instead, we chose two distinct pharmacological options that might rescue the downstream phenotypes, including apoptotic cell death arising from autophagy deficiency. One of the compounds is nicotinamide mononucleotide (NMN), which is a bioavailable NAD^+^ precursor (Yoshino et al., 2018). We have shown that loss of autophagy mediates cell death by depletion of NAD^+^ and NADH levels, whereas NMN can restore total NAD pool, rescue deleterious cellular phenotypes, and improve survival in autophagy-deficient cells (Kataura et al., 2022; Sun et al., 2023). This forms the rationale for testing the ability of NMN to rescue the cellular and functional phenotypes of *ATG5*^−/−^ LG-like organoids. Another compound we selected is melatonin, since it is produced in various ocular tissues including LG, retina, lens, iris, and ciliary body (Yu et al., 2021).

**Figure 5:**
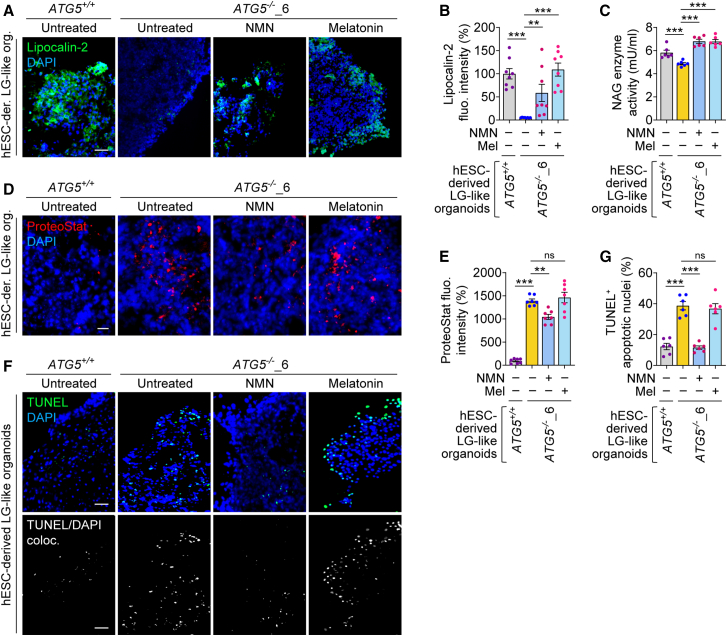
Pharmacological rescue of functional and cellular phenotypes in autophagy-deficient hESC-derived LG-like organoids (A–G) Immunofluorescence images of lipocalin-2 (A) and quantification of fluorescence intensity (B); NAG enzyme activity (C); fluorescence images of ProteoStat staining (D) and quantification of fluorescence intensity (E); and fluorescence images of TUNEL staining (F) and quantification of TUNEL^+^ apoptotic nuclei (G) in *ATG5*^+/+^ and *ATG5*^−/−^_6 hESC-derived LG-like organoids, where *ATG5*^−/−^ organoids were treated with or without 1 mM NMN or 1 μM melatonin (Mel) for 48 h. Graphical data are mean ± SEM of *n* = 6–8 experimental replicates from 3 independent experiments; *p* values calculated by one-way ANOVA followed by multiple comparisons with the two-stage linear step-up procedure of Benjamini, Krieger, and Yekutieli (B, C, E, and G). ^∗∗^*p* < 0.01; ^∗∗∗^*p* < 0.001; ns, non-significant. Scale bar: 50 μm (A, D, and F).

We first analyzed tear proteins in LG-like organoids. Both NMN and melatonin increased lipocalin-2 expression and NAG enzyme activity (associated with lysozyme secretion) in *ATG5*^−/−^ LG-like organoids (Figures 5A–5C), suggesting an improvement in the secretory function. We next analyzed the effects of these compounds on autophagy-related cellular phenotypes. NMN, but not melatonin, partially reduced aggresomes (ProteoStat signal) in *ATG5*^−/−^ LG-like organoids (Figures 5D and 5E). Concomitantly, NMN reduced cell death (TUNEL^+^ apoptotic nuclei) in *ATG5*^−/−^ LG-like organoids to levels comparable to those observed in *ATG5*^*+/+*^ organoids; however, melatonin had no effect (Figures 5F and 5G). The amelioration of cellular defects by NMN is likely due to its cytoprotective effects that we have shown in the context of autophagy deficiency (Kataura et al., 2022; Sun et al., 2023). These data highlight the potential of NMN in rescuing cellular and functional phenotypes in autophagy-deficient LG-like organoids, while melatonin is only effective for improving secretory function.

### NMN confers cytoprotection during autophagy deficiency by restoring protein and mitochondrial homeostasis

**Figure 6:**
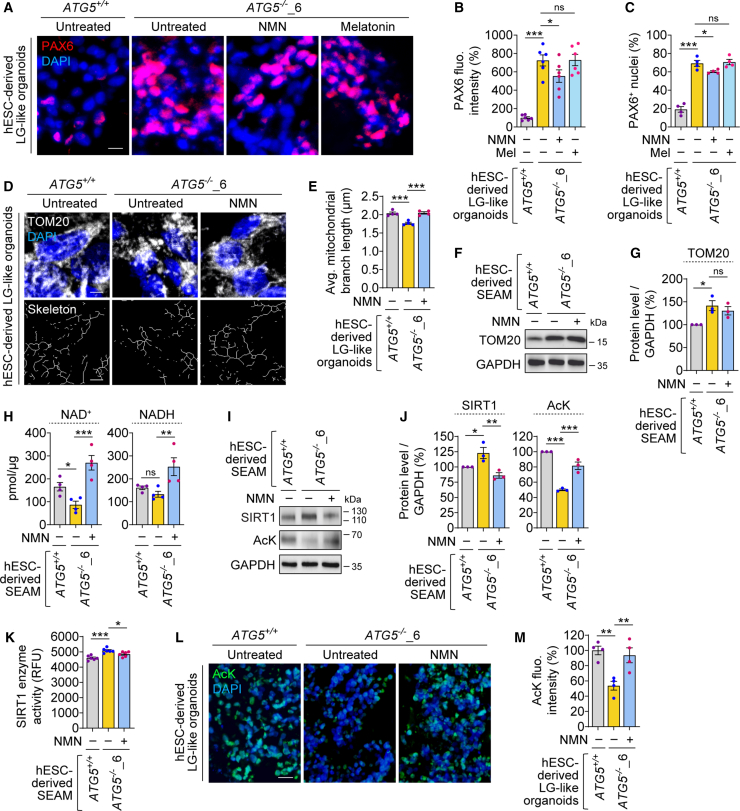
NMN restores NAD^+^, PAX6 levels, mitochondrial homeostasis, and SIRT1 activity in autophagy-deficient hESC-derived SEAM and LG-like organoids (A–M) Immunofluorescence images of PAX6 (A) and quantification of fluorescence intensity (B) and PAX6^+^ nuclei (C); immunofluorescence and skeleton images of TOM20 (D) and quantification of average mitochondrial branch length (E); immunoblotting (F) and densitometric (G) analyses of TOM20 relative to GAPDH; measurement of NAD^+^ and NADH levels (H); immunoblotting (I) and densitometric (J) analyses of SIRT1 and acetylated lysine (AcK) relative to GAPDH; SIRT1 enzyme activity (K); and fluorescence images of AcK (D) and quantification of fluorescence intensity (M) in *ATG5*^+/+^ and *ATG5*^−/−^_6 hESC-derived SEAM or LG-like organoids, where *ATG5*^−/−^ SEAM and LG-like organoids were treated with or without 1 mM NMN or 1 μM melatonin (Mel) for 48 h, as indicated. Graphical data are mean ± SEM of *n* = 3–6 experimental replicates from 3 independent experiments; *p* values calculated by one-way ANOVA followed by multiple comparisons with a two-stage linear step-up procedure of Benjamini, Krieger and Yekutieli (B, C, E, G, H, J, K, M). ^∗^*p* < 0.05; ^∗∗^*p* < 0.01; ^∗∗∗^*p* < 0.001; ns, non-significant. Scale bar: 10 μm (D), 50 μm (A, L).

We further investigated the effects of NMN and melatonin on PAX6 levels, which were elevated in *ATG5*^−/−^ LG-like organoids (Figures 4C, 4D, and 6A–6C). Interestingly, NMN had a moderate effect in reducing PAX6 accumulation in *ATG5*^−/−^ LG-like organoids, while melatonin had no effect (Figures 6A–6C). A possible mechanism underlying the effect of NMN on PAX6 may involve the impact of NAD^+^ augmentation in improving mitochondrial homeostasis, which is interconnected with cellular proteostasis (Sun et al., 2023; Wang et al., 2022). We showed in *ATG5*^−/−^ hESC-derived neurons that NMN reduces the buildup of aggresomes, concomitant with restoring mitochondrial homeostasis (Sun et al., 2023). Since NMN also lowered aggresomes in *ATG5*^−/−^ LG-like organoids (Figures 5D and 5E), we studied if it influences mitochondrial homeostasis. We found that reduced mitochondrial branch length in *ATG5*^−/−^ LG-like organoids was restored by NMN to a level comparable to that of wild-type LG-like organoids (Figures 6D and 6E). This was not due to clearance of damaged mitochondria because NMN did not affect mitochondrial load in the autophagy-deficient condition, as analyzed by TOM20 (outer mitochondrial membrane protein) levels (Figures 6F and 6G).

We next investigated whether NMN influences mitochondrial homeostasis potentially through modulation of sirtuin (SIRT) activity (Imai and Guarente, 2014). SIRTs are a family of NAD^+^-dependent deacetylases that regulate gene expression and protein stabilization by removing acetyl groups from proteins including histones and play key roles in protein and mitochondrial homeostasis (Wu et al., 2022). It is possible that the maintenance of this homeostatic balance by NMN, through increasing NAD^+^ and NADH levels (Figure 6H), also influences PAX6 in the context of autophagy deficiency. To investigate the potential mechanism underlying this effect, we examined the role of NAD^+^-dependent SIRT1 activity. The levels of SIRT1, a member of the sirtuin family, were elevated in *ATG5*^−/−^ SEAM (Figures 6I and 6J), likely because SIRT1 is a nuclear autophagy substrate (Xu et al., 2020). SIRT1 activity was also increased, as evident from its higher enzymatic activity (Figure 6K) and reduction in protein acetylation of acetylated lysine (AcK) in *ATG5*^−/−^ SEAM and LG-like organoids (Figures 6I, 6J, 6L, and 6M). As we reported in autophagy-deficient cells (Kataura et al., 2022; Sun et al., 2023), SIRT1 hyperactivation could be driving NAD^+^ depletion during loss of autophagy (Figures 2J and 6H–6M). Interestingly, NMN treatment normalized SIRT1 levels and its activity in *ATG5*^−/−^ SEAM and LG-like organoids to those of wild-type levels (Figures 6I–6M), suggesting that SIRT1 hyperactivation driven by autophagy deficiency can be restored through NAD^+^ supplementation. Collectively, these findings suggest that NMN improves overall cellular homeostasis and survival during stress conditions, likely through restoration of the NAD pool, SIRT1 activity, mitochondrial homeostasis, and proteostasis, which may potentially facilitate the recovery of PAX6 levels.

## Discussion

**Figure 7:**
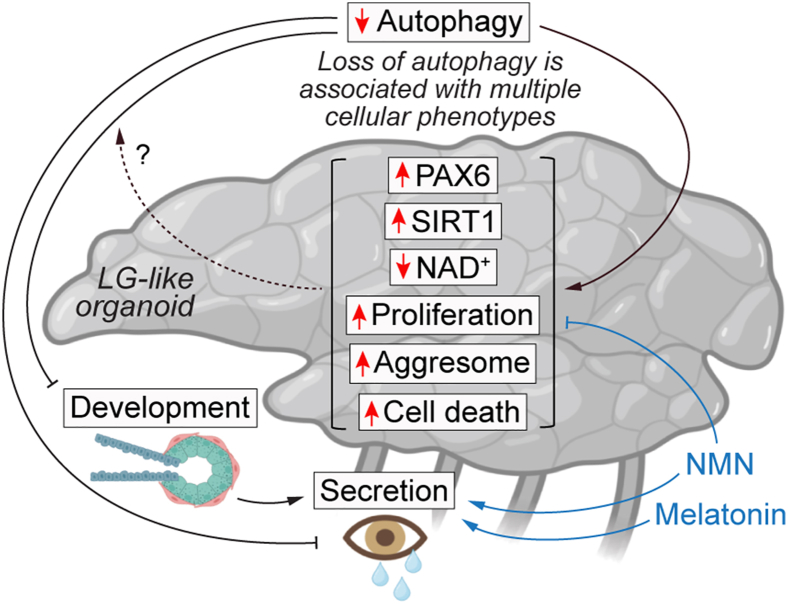
Schematic illustration of cellular and functional phenotypes of autophagy-deficient LG-like organoids that could be restored by pharmacological interventions LG-like organoids with loss of autophagy are associated with improper development and secretory function. At a cellular level, autophagy-deficient LG-like organoids exhibit elevation in PAX6 levels, SIRT1 activity, proliferation, aggresomes, and cell death and depletion of NAD^+^ levels. Pharmacological intervention with NMN and melatonin rescues the secretory function, while NMN restores multiple cellular phenotypes and improves cell viability in autophagy-deficient LG-like organoids.

In summary, we utilized a genetic knockout hESC model of autophagy deficiency (Sun et al., 2023) to generate SEAM and LG-like organoids to demonstrate that autophagy is necessary for LG maintenance and functionality. LG cell composition, involving acinar/ductal cells and MECs, is crucial for its development and aqueous tear film formation. Particularly, MECs are essential for polarization of acini, normal morphogenesis, gland maintenance, and secretion, by supporting the differentiation of acinar cells via secretion of cytokines and growth factors (Basova et al., 2020). We found that loss of autophagy decreased the expression of LG-related markers, tear protein secretion, and enzyme activity. Transcriptomics analysis revealed perturbations in *ATG5*^−/−^ SEAM in processes related to eye and visual system development, gland morphogenesis, and epithelial cell differentiation that are essential for LG development. These findings suggest that loss of autophagy leads to improper LG development and function (Figure 7).

Autophagy is a vital homeostatic process for maintaining cellular proteostasis and survival, whereas its malfunction contributes to protein aggregation and cell death in age-related degenerative diseases (Aman et al., 2021). We have shown that abrogation of autophagy mediates cytotoxicity by depletion of NAD levels (Kataura et al., 2022; Sun et al., 2023). We found *ATG5*^−/−^ SEAM and LG-like organoids exhibited aggresome accumulation, NAD^+^ depletion, and cell death, along with elevated cell proliferation. Transcriptomics analysis also showed perturbations in biological processes linked to these phenotypes. Increased apoptosis and proliferation have been shown to cause eye defects in zebrafish (Shohayeb and Cooper, 2023). Thus, it is plausible that higher cell death and proliferation due to autophagy deficiency might affect LG development (Figure 7).

Interestingly, we found PAX6 to be an autophagy substrate that is accumulated in *ATG5*^−/−^ SEAM and LG-like organoids, along with deregulation of its target gene expression. Colocalization of the nuclear protein PAX6 with autophagosomes and lysosomes in the proximity of the nucleus in wild-type cells indicates that PAX6 could be degraded by nucleophagy. Although PAX6 has a role in eye and brain development (Georgala et al., 2011; Kozmik, 2005), its importance in LG maintenance is poorly understood. In zebrafish, Pax6 plays a divergent role in cell fate decision such as self-renewal vs. proliferation of neural stem cells in a dosage- and context-dependent manner (Thummel et al., 2010), while *Pax6* knockout mouse LG organoids were associated with loss of expression of secretion-related genes and tear products (Bannier-Hélaouët et al., 2021). Additionally, differential expression of PAX6 and its activity are implicated in lineage-specific differentiation of ocular surface epithelium cells (Kamuro et al., 2025). In the context of *ATG5*^−/−^ LG-like organoids, we found that NMN-mediated restoration of cellular phenotypes involving aggresome accumulation, mitochondrial fragmentation, SIRT1 hyperactivation, and cell death correlated with normalization of PAX6 to wild-type levels (Figure 7). Growing evidence suggests that boosting NAD^+^ levels by NAD^+^ precursor supplementation improves mitochondrial homeostasis, which is closely linked to cellular proteostasis (Sun et al., 2023; Wang et al., 2022), and this NMN-mediated interplay may potentially restore PAX6 levels during loss of autophagy. Our study raises the possibility that an optimal level of PAX6 is important for LG development, whereas its imbalance might disrupt this process.

Lacrimal secretion is primarily regulated by phospholipase C activation, generating inositol 1,4,5-trisphosphate and triggering endoplasmic reticulum Ca^2+^ release that influences downstream pathways (Putney and Bird, 2014). Melatonin receptors can modulate Ca^2+^ channel activity to trigger Ca^2+^ release (Huete-Toral et al., 2015), while boosting NAD^+^ levels can also indirectly affect Ca^2+^ channels (Rissiek et al., 2015). This might explain how melatonin and NMN affected secretion in *ATG5*^−/−^ LG-like organoids. Moreover, we have shown that elevating NAD^+^ levels improved proteostasis, mitochondrial homeostasis, and the viability of autophagy-deficient cells and organisms (Kataura et al., 2022; Sun et al., 2023), effects that were also found in *ATG5*^−/−^ LG-like organoids after NMN treatment (Figure 7). These findings highlight the potential therapeutic benefits of these agents in DED, warranting further investigation in ocular disease models.

The autophagy-deficient LG organoid platform provides a tool to understand the role of autophagy in LG and identify potential agents for DED treatment. We have recently shown that ocular organoids could be pertinent with organ-on-chip platforms that mimic tissue shear via microfluidics (Koçak et al., 2024). A similar approach can be undertaken using our genetic hESC model to study the physiological role of autophagy in other glands with similar developmental patterns, including the mammary gland, salivary gland, pancreas, and lungs, as well as other ocular cells derived from SEAM.

### Limitations of the study

This study focuses on the role of autophagy in LG differentiation using an *ATG5*^−/−^ hESC model of autophagy deficiency. However, *in vivo* validation was not possible because, currently, there are no animal models with LG-specific conditional *Atg* knockout. Moreover, it was beyond the scope of this study to knock out another *ATG* gene for generating an additional autophagy-deficient hESC line, which is currently unavailable; instead, autophagy inhibitors were used. Studying if autophagy induction would promote LG differentiation in wild-type cells would require making a stable line with ATG overexpression because the known autophagy enhancers target upstream signaling pathways and, thus, are not ideal for long-term treatment during the differentiation period. Demonstrating PAX6-LC3 interaction by endogenous immunoprecipitation was technically challenging due to the large amount of organoid material required; instead, PLA was performed.

## Methods

### hESC culture and SEAM differentiation

*ATG5*^*+/+*^ and *ATG5*^−/−^ hESC lines were cultured feeder-free, as previously described (Sun et al., 2023). The differentiation of ocular cell lineages from hESC lines was performed according to the previously reported SEAM formation method (Hayashi et al., 2022). SEAM differentiation was done for 12 weeks until cell sorting for generating 3D LG-like organoids.

### Generation of 3D LG-like organoids from hESC-derived SEAM

Cell sorting of SSEA-4^+^/ITGB4^+^ cells from hESC-derived SEAM was performed to obtain ocular surface ectodermal cells from ocular cell lineages using flow cytometry (Table S1). Sorted SSEA-4^+^/ITGB4^+^ cells were embedded in 50% (v/v) growth factor-reduced Matrigel and LG culture medium for 30 days to generate LG-like organoids.

### Immunoblotting, immunofluorescence and gene expression analysis

Markers for cellular identity and functionality were analyzed by immunoblotting, immunofluorescence, and qPCR. Primary/secondary antibodies and gene primers are listed in Tables S2–S4.

### Analyses of cellular phenotypes

Analyses of autophagy, cell death, cell proliferation, mitochondrial branch length, and aggresomes were respectively done by staining for LC3 and p62, cleaved caspase-3 and TUNEL, Ki-67, TOM20, and ProteoStat. NAG and SIRT1 enzyme activity, and NAD^+^ and NADH levels, were measured by assay kits.

### Identification of PAX6 as autophagy substrate

Putative LIR motifs within PAX6 were identified *in silico* using the iLIR Autophagy Database. Multiple sequence alignment was conducted to assess their conservation among species. Colocalization of PAX6 with LC3 and LAMP1 by immunofluorescence was measured using Pearson’s correlation coefficient and PLA. Autophagic degradation of PAX6 was analyzed by immunoblotting.

### Transcriptomics data analysis

For identification of DEGs, we used the DESeq2 package. Accuracy of fold change estimation was done by apeglm. Threshold for differential expression was set considering Benjamini-Hochberg *p* adj. value < 0.05, |Log2(foldchange)| > 1 as significant. PCA and heatmap of RNA-seq expression Z-scores were plotted using ggplot2. GO analysis was performed using g:Profiler with g:SCS multiple testing correction method; *p* values <0.05. Bubble plots were generated using SRplot.

### Statistical analysis

Graphical data are mean ± s.e.m from *n* ≥ 3 experimental replicates from 3 independent experiments, using GraphPad Prism. *p* values were determined by unpaired two-tailed Student’s *t* test or one-way ANOVA followed by multiple comparisons with the two-stage linear step-up procedure of Benjamini, Krieger, and Yekutieli. For transcriptomics analysis (*n* = 3 experimental replicates), the threshold for differential gene expression was set, considering Benjamini-Hochberg *p* adj. value < 0.05, |Log2(foldchange)| > 1 as significant. GO: Biological Process terms were selected based on corrected *p* value < 0.05, using the g:SCS multiple testing correction method. ^∗∗∗^*p* < 0.001; ^∗∗^*p* < 0.01; ^∗^*p* < 0.05; ns, non-significant.

Detailed methodologies are provided in supplemental methods in supplemental information.

## Resource availability

### Lead contact

Requests for further information and resources should be directed to and will be fulfilled by the lead contact, S. Sarkar (s.sarkar@bham.ac.uk).

### Materials availability

WIBR3 parental hESC line, generated by Jaenisch lab at Whitehead Institute, was used at University of Birmingham under material transfer agreements, UBMTA 15–0593 and 15–0595.

### Data and code availability

Data of results are presented in the main manuscript and supplemental information. Transcriptomics data were deposited in the GEO database; GEO: GSE280811. This paper does not report any original code.

## Acknowledgments

This work was supported by grants from 10.13039/501100000265Medical Research Council (MRC; MR/Z504488/1), 10.13039/501100000317Action Medical Research/LifeArc (GN3049), LifeArc (Philanthropic Fund P2019-0004, Pathfinder Award), 10.13039/100010269Wellcome Trust (109626/Z/15/Z), and Birmingham Fellowship to S.S.; MRC (MR/P007732/1) to T.B.; TÜBİTAK-BİDEB 2214A Fellowship to G.K. and S.S.; YÖK 100/2000 PhD Scholarship and TÜBİTAK-BİDEB 2211A Fellowship to G.K.; TÜBİTAK (121C313) and TÜSEB Group A (28778) projects to S.G.; and BBSRC- and UoB-funded MIBTP PhD Studentship (BB/T00746X/1) to M.E.K. and S.S. We thank N. Mizushima, Y. Kanda, J. Barlow, and V. Korolchuk for assistance/feedback. S.S. is also a Former Fellow (life) at Hughes Hall, University of Cambridge, UK.

## Author contributions

G.K. and S.S. conceptualized the project; G.K., M.E.K., L.F.A., C.S., M.C., A.G., M.K., M.Z., E.S., A.A., B.Y., A.M., and S.G. performed experiments, provided reagents, and/or analyzed data; S.S., G.K., S.G., M.E.K., and T.B. acquired funding; G.K., M.E.K., and S.S. prepared the figures; G.K. and S.S. wrote the manuscript, and all authors contributed to and approved it.

## Declaration of interests

S.S. and T.B. are scientific advisors and E.S. is founder and CEO of NMN Bio Ltd, UK. M.K. and B.Y. are employees of SZAOMICS Biotechnology R&D, Türkiye.

## Supplemental information

Document S1. Figures S1–S4, Tables S1–S4, and supplemental methods

Document S2. Article plus supplemental information
