# Supplementary material for: Autophagy is required for the development and functionality of lacrimal gland-like organoids
Source: Stem Cell Reports. 2025 Dec 18;21(1):102744. doi: 10.1016/j.stemcr.2025.102744 (PMC12925961; doi:10.1016/j.stemcr.2025.102744)
Supplement: Document S1. Figures S1–S4, Tables S1–S4, and supplemental methods [file mmc1.pdf]

Stem Cell Reports, Volume *21*

Supplemental Information

Autophagy is required for the development and functionality of lacrimal gland-like organoids

Gamze Kocak, Miriam E. Korsgen, Leticia F. Amores, Congxin Sun, Merve Ceylan, Asmaa Ghazwani, Merve Kandirici, Malgorzata Zatyka, Elena Seranova, Animesh Acharjee, Timothy Barrett, Bayram Yuksel, Adil Mardinoglu, Sinan Güven, and Sovan Sarkar

SUPPLEMENTAL FIGURES

Figure S1

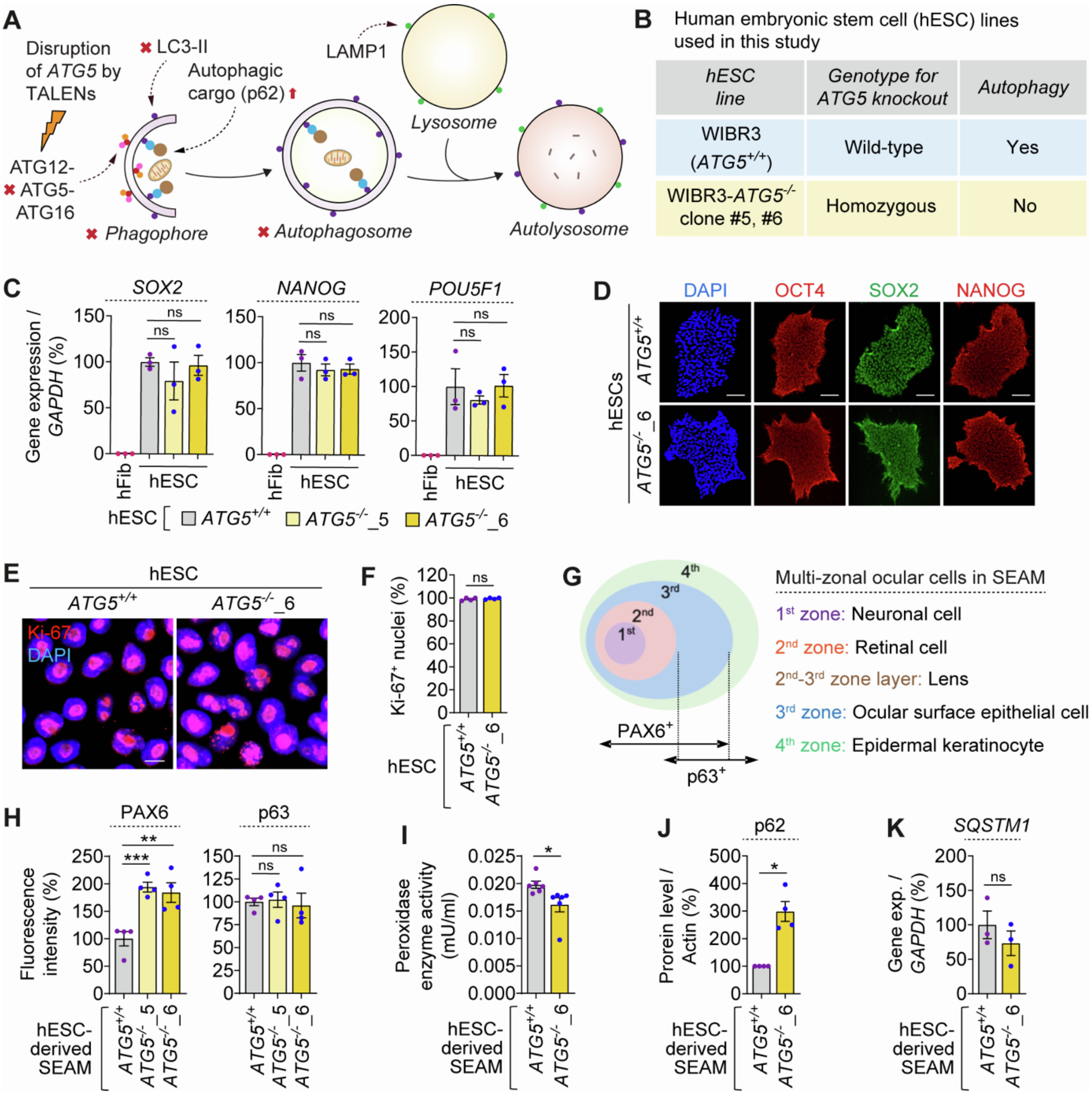

Figure S1. Characterisation of cellular identity and phenotypes in hESCs and hESC-derived SEAM (related to Figure 1).
(A) Schematic illustration of the impact on autophagy due to *ATG5* gene disruption by TALENs. Red cross denotes absence or loss, and red up arrow denotes accumulation.
(B) List of wild-type and autophagy-deficient hESC lines used in this study.
(C, D) Gene expression analysis of *SOX2*, *NANOG* and *POU5F1* relative to *GAPDH* (C), and immunofluorescence images of SOX2, OCT4 and NANOG (D), in ATG5$^{+/+}$, ATG5$^{-/-}$_5 and ATG5$^{-/-}$_6 hESCs. Human fibroblasts (hFib) were used as negative control (C).
(E, F) Immunofluorescence images of Ki-67 (E) and quantification of Ki-67^{+} nuclei (F) in *ATG5*$^{+/+}$ and *ATG5*$^{-/-}$_6 hESCs.
(G) Schematic illustration of five distinct zones of multi-zonal ocular cells in SEAM. These zones comprise neural crest zone 1, retinal-like cells zone 2, lens-like cells zone 2-3, ocular surface epithelial-like cells zone 3, and epidermal keratinocytes zone 4. Zone 3 consists of the ocular surface epithelial cells, co-expressing PAX6 and p63.

(H) Quantification of fluorescence intensity of PAX6 and p63 in *ATG5*$^{+/+}$, *ATG5*$^{-/-}$_5 and *ATG5*$^{-/-}$_6 hESC-derived SEAM (related to immunofluorescence images in Figure 1C).
(I–K) Peroxidase enzyme activity (I), densitometric analysis of p62 relative to Actin (related to immunoblot in Figure 1L) (J), and gene expression analysis of *SQSTM1* relative to *GAPDH* (K) in *ATG5*$^{+/+}$ and *ATG5*$^{-/-}$_6 hESC-derived SEAM.

Graphical data are mean ± SEM of n = 3–6 experimental replicates from 3 independent experiments (C, F, H–K). P values were calculated by unpaired two-tailed Student's t-test (F, I–K), or one-way ANOVA followed by multiple comparisons with a two-stage linear step-up procedure of Benjamini, Krieger and Yekutieli (C, H). *P<0.05; **P<0.01; ***P<0.001; ns, non-significant. Scale bar, 50 μm (D, E).

Figure S2

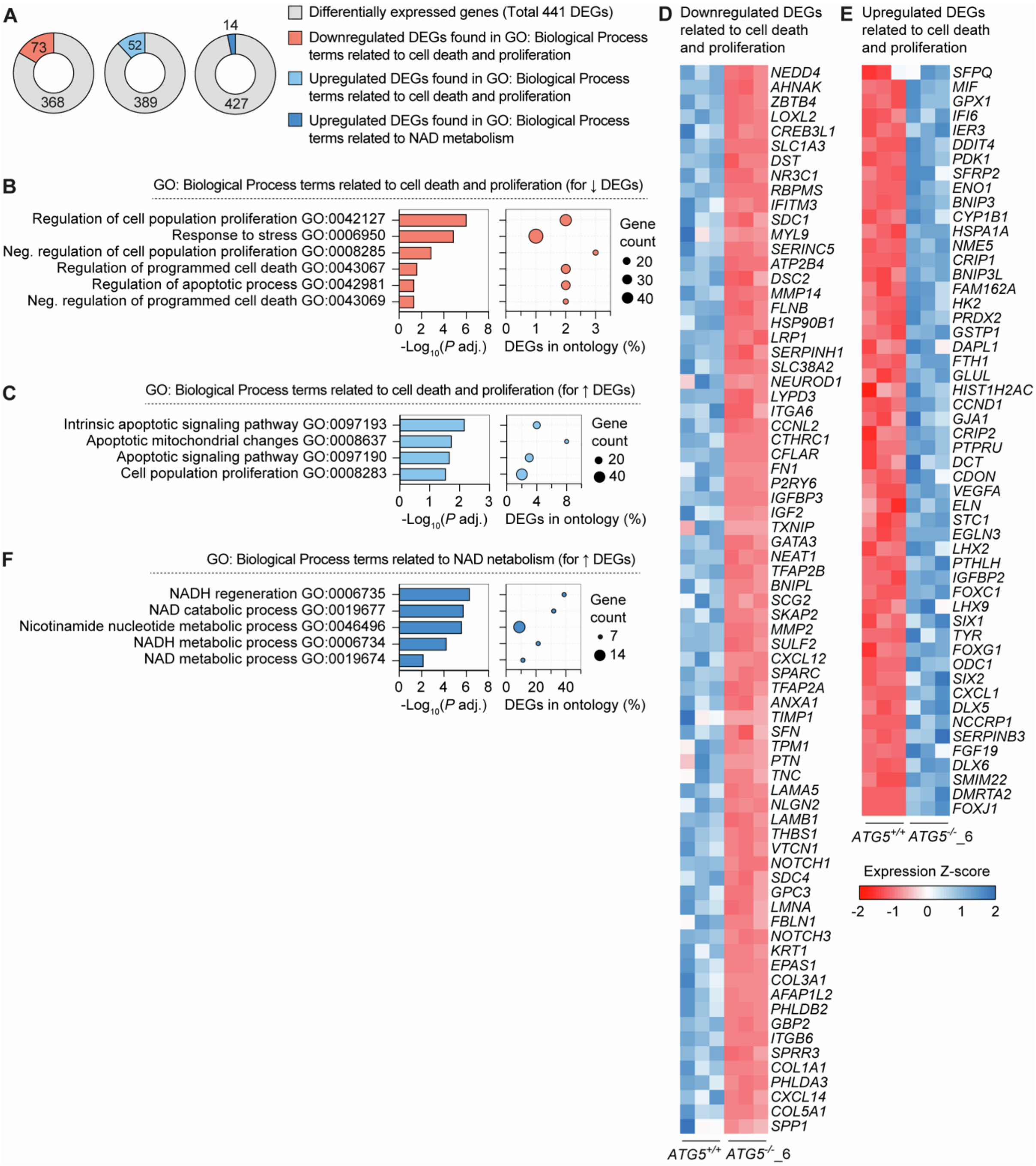

Figure S2. Perturbations in gene expression in autophagy-deficient hESC-derived SEAM (related to Figure 2).

(A–F) Pie charts (A), bar and dot plots (B, C, F), and heatmaps (D, E) of downregulated or upregulated differentially regulated genes (DEGs) enriched in Gene Ontology (GO): Biological Process terms related to cell death and proliferation (A–E) or NAD metabolism (A, F) in *ATG5*$^{-/-}$_6 hESC-derived SEAM compared to *ATG5*$^{+/+}$ hESC-derived SEAM.

For transcriptomics analysis (n = 3 experimental replicates from 3 independent experiments), threshold for differential gene expression was set, considering the Benjamini-Hochberg *P* adj. value < 0.05, |Log2(foldchange)| > 1 as significant. GO: Biological Process terms were selected as per their *P* value < 0.05 corrected with g:SCS multiple testing correction method. The dot sizes indicate the number of DEGs (B, C, F).

Figure S3

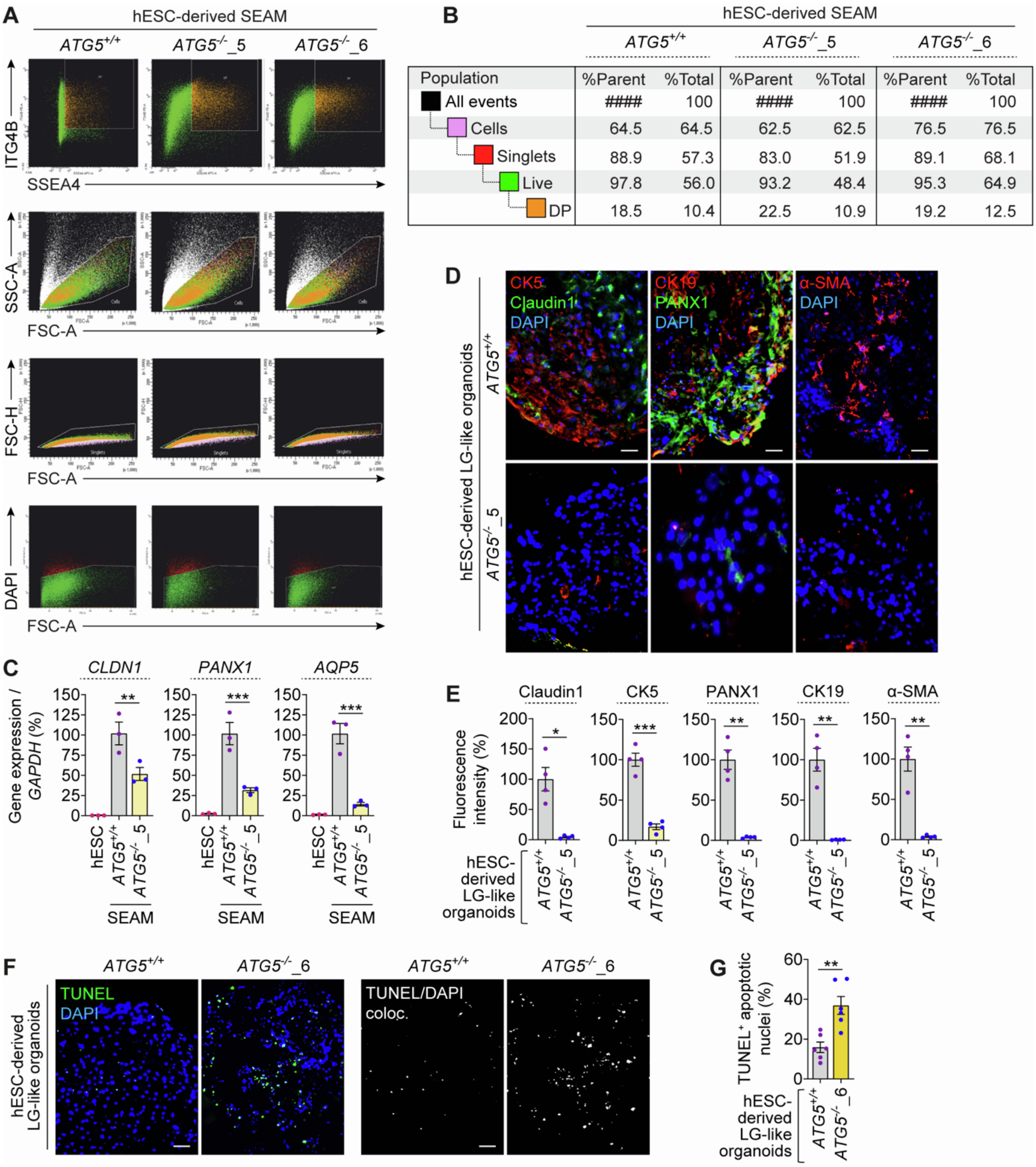

Figure S3. Improper differentiation and cell death in autophagy-deficient hESC-derived SEAM and LG-like organoids (related to Figure 3).

(A, B) Flow cytometry gating strategy (A) and percentage for all events (B) for SSEA4/ITG4B double-positive cell sorting of *ATG5*$^{+/+}$, *ATG5*$^{-/-}$_5 and *ATG5*$^{-/-}$_6 hESC-derived SEAM.

(C–E) Gene expression analyses of *CLDN1*, *PANX1*, and *AQP5* relative to *GAPDH* (C), immunofluorescence images of Claudin1, CK5, PANX1, CK19, and α-SMA (D) and quantification of fluorescence intensity (E) in *ATG5*$^{+/+}$ and *ATG5*$^{-/-}$_5 hESC-derived SEAM (C) or LG-like organoids (D, E). Wild-type hESCs were used as negative control (C).

(F, G) Fluorescence images of TUNEL staining (F) and quantification of TUNEL^{+} apoptotic nuclei (G) in the *ATG5*$^{+/+}$ and *ATG5*$^{-/-}$_6 hESC-derived LG-like organoids.

Graphical data are mean ± SEM of n = 3–6 experimental replicates from 3 independent experiments (C, E, G). *P* values were calculated by unpaired two-tailed Student's t-test (E, G), or one-way ANOVA

followed by multiple comparisons with a two-stage linear step-up procedure of Benjamini, Krieger and Yekutieli (C). *P<0.05; **P<0.01; ***P<0.001. Scale bar, 50 μm (D, F).

Figure S4

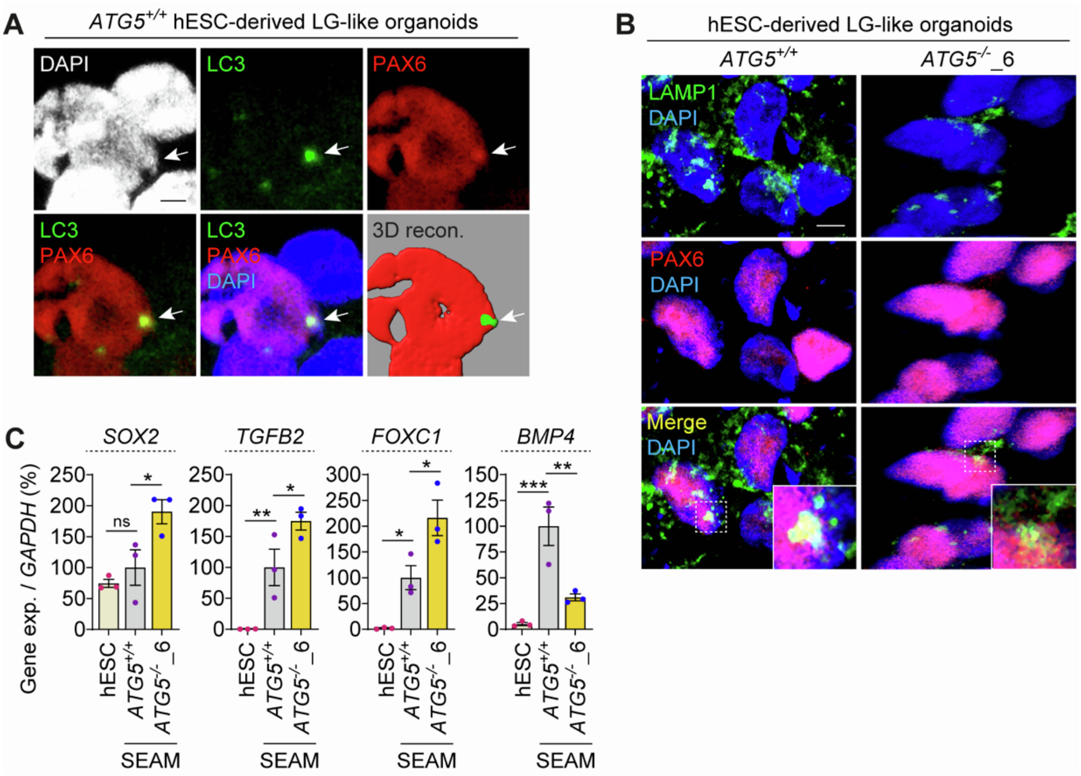

Figure S4. PAX6 localization with autophagic vesicles and its target gene expression in hESC-derived SEAM and LG-like organoids (related to Figure 4).

(A) 3D reconstruction of PAX6–LC3 colocalization after co-immunostaining in *ATG5*$^{+/+}$ hESC-derived LG-like organoids.

(B) Immunofluorescence images of PAX6 and LAMP1 in *ATG5*$^{+/+}$ and *ATG5*$^{-/-}$_6 hESC-derived LG-like organoids.

(C) Gene expression analysis of *SOX2*, *TGFB2*, *FOXC1* and *BMP4* (PAX6 target genes) relative to *GAPDH* in in *ATG5*$^{+/+}$ and *ATG5*$^{-/-}$_6 hESC-derived SEAM; wild-type hESCs were used as negative control.

Graphical data are mean ± SEM of n = 3 experimental replicates from 3 independent experiments (C). P values were calculated by one-way ANOVA followed by multiple comparisons with a two-stage linear step-up procedure of Benjamini, Krieger and Yekutieli (C). *P<0.05; **P<0.01; ***P<0.001; ns, non-significant. Scale bar, 10 µm (B) or 20 µm (A).

SUPPLEMENTAL TABLES

Table S1

Antigen	Host species	Source	Catalogue number	Dilution
**Primary antibodies for flow cytometry**				
SSEA4-AF647	Mouse	Biolegend	330407	1:20
ITGB4-PE	Mouse	Biolegend	327807	1:20

Table S1. List of primary antibodies for cell sorting by flow cytometry (related to Figure 3 and S3).
Details of antibodies used in hESC-derived SEAM for cell sorting by flow cytometry.

Table S2

Antigen	Host species	Source	Catalogue number	Dilution
**Primary antibodies for immunoblotting**				
GAPDH	Mouse	Sigma-Aldrich	G8795	1:5000
Actin	Rabbit	Sigma-Aldrich	A2066	1:4000
PAX6	Mouse	Santa Cruz Biotechnology	sc-81649	1:1000
p62	Mouse	Proteintech	66184-1-Ig	1:3000
ATG5	Mouse	Santa Cruz Biotechnology	sc-133158	1:500
LC3B	Rabbit	Proteintech	14600-1-AP	1:2000
α-SMA	Mouse	Cell Signaling Technology	48938S	1:1000
CK5	Mouse	Thermo Scientific	MA517057	1:1000
CK19	Mouse	Cell Signaling Technology	4558S	1:1000
TOM20	Mouse	Santa Cruz Biotechnology	sc-17764	1:5000
Acetylated lysine	Rabbit	Cell Signaling Technology	9441S	1:1000
SIRT1	Mouse	Proteintech	60303-1-IG	1:5000
**Secondary antibodies for immunoblotting**				
Anti-rabbit IgG, HRP-conjugated	Goat	Dako	P0448	1:10000
Anti-mouse IgG, HRP-conjugated	Rabbit	Dako	P0161	1:10000

Table S2. List of primary and secondary antibodies for immunoblotting analysis (related to Figure 1, 4 and 6).
Details of antibodies used in hESCs and hESC-derived SEAM for immunoblotting analysis.

Table S3

Antigen	Host species	Source	Catalogue number	Dilution
**Primary antibodies for immunofluorescence**				
Ki-67	Mouse	Cell Signaling Technology	9449	1:400
NANOG	Goat	R&D Systems	AF1997	1:100
OCT-3/4	Goat	R&D Systems	AF1759	1:100
SOX2	Mouse	R&D Systems	AF2018	1:100
P63	Goat	R&D Systems.	AF1916	1:200
PAX6	Rabbit	Biolegend	901301	1:100
CD44	Mouse	Santa Cruz Biotechnology	sc-7297	1:200
LC3B	Mouse	Nanotools	0231-100	1:200
LC3B	Rabbit	Proteintech	14600-1-AP	1:300
p62	Mouse	Proteintech	66184-1-Ig	1:300
α-SMA	Mouse	Cell Signaling Technology	48938S	1:200
CK5	Mouse	Thermo Scientific	MA517057	1:200
PANX1	Rabbit	Sigma-Aldrich	HPA016930	1:100
Claudin1	Rabbit	Cell Signaling Technology	13995S	1:200
AQP5	Rabbit	Bioss	bs-1554R	1:200
Lactoferrin	Rabbit	ABCAM	ab109216	1:200
Lipocalin2	Rabbit	ABCAM	ab41105	1:200
Caspase-3	Mouse	Novus	NB100-56709	1:500
CK19	Mouse	Cell Signaling Technology	4558S	1:200
Acelylated lysine	Rabbit	Cell Signaling Technology	9441S	1:200
TOM20	Mouse	Santa Cruz Biotechnology	sc-17764	1:100
LAMP1	Mouse	Cell Signaling Technology	15665S	1:200
**Secondary antibodies for immunofluorescence**				
Anti-mouse IgG (H+L), Alexa Fluor 594	Donkey	Thermo Fisher Scientific	A-21203	1:1000
Anti-rabbit IgG (H+L), Alexa Fluor 488	Donkey	Thermo Fisher Scientific	A-21206	1:1000
Anti-mouse IgG (H+L), Alexa Fluor 488	Donkey	Thermo Fisher Scientific	A-21202	1:1000
Anti-rabbit IgG (H+L), Alexa Fluor 594	Donkey	Thermo Fisher Scientific	A-21207	1:1000
Anti-goat IgG (H+L), Alexa Fluor 647	Donkey	Thermo Fisher Scientific	A-21447	1:1000

Table S3. List of primary and secondary antibodies for immunofluorescence analysis (related to Figure 1–6, S1, S3 and S4).
Details of antibodies used in hESCs and hESC-derived SEAM and LG-like organoids for immunofluorescence analysis.

Table S4

Gene	Species	Primer direction	Primer sequence	Source
**For gene expression analysis using SYBR Green real-time PCR**				
AQP5	Human	Forward	TACGGTGTGGCACCGCTCAATG	Merck
		Reverse	AGTCAGTGGAGGCGAAGATGCA	
KRT19	Human	Forward	AGCTAGAGGTGAAGATCCGCGA	Merck
		Reverse	GCAGGACAATCCTGGAGTTCTC	
PANX1	Human	Forward	GCTGTGGACAAGATGGTCACGT	Merck
		Reverse	CGCCAGGAGAAAGAACTTGGAG	
CLDN1	Human	Forward	GTCTTTGACTCCTTGCTGAATCTG	Merck
		Reverse	CACCTCATCGTCTTCCAAGCAC	
ACTA2	Human	Forward	CTATGCCTCTGGACGCACAACT	Merck
		Reverse	CAGATCCAGACGCATGATGGCA	
PAX6	Human	Forward	ACACACATGAACAGTCAGCCA	Merck
		Reverse	TCAGGTTCACTTCCGGGAAC	
POU5F1	Human	Forward	TCTATTTGGGAAGGTATTC	Merck
		Reverse	TTGCATATCTCCTGAAGA	
SOX2	Human	Forward	CGGACAGCGAACTGGAGG	Merck
		Reverse	CTGTTTCTTACTCTCCTCTTTTGC	
NANOG	Human	Forward	TGTGTACTCAATGATAGATT	Merck
		Reverse	GTCTTCACCTGTTTGTAG	
TGFB2	Human	Forward	*AAGAAGCGTGCTTTGGATGCGG*	Merck
		Reverse	*ATGCTCCAGCACAGAAGTTGGC*	
FOXC1	Human	Forward	*AGAAGGACAGGCTGCACCTCAA*	Merck
		Reverse	*GTTCTCGGTCTTGATGTCCTGG*	
BMP4	Human	Forward	*CTGGTCTTGAGTATCCTGAGCG*	Merck
		Reverse	*TCACCTCGTTCTCAGGGATGCT*	
GAPDH	Human	Forward	GTCTCCTCTGACTTCAACAGCG	Merck
		Reverse	ACCACCCTGTTGCTGTAGCCAA	

Gene	Species	Assay ID	Source
**For gene expression analysis using Taqman Assay real-time PCR**			
SQSTM1	Human	Hs01061917_g1	Applied Biosystem
ACTB	Human	Hs01060665_g1	Applied Biosystem

Table S4. List of primers and assays for gene expression analysis (related to Figure 1, 4, S1, S3 and S4).
Details of primers and assays used for gene expression analysis by SYBR Green real-time PCR master mix or Taqman assay in hESCs and hESC-derived SEAM.

SUPPLEMENTAL METHODS

Human embryonic stem cell culture

The WIBR3 wild-type (*ATG5*$^{+/+}$) human embryonic stem cell (hESC) line [female line of European origin; NIH approval number NIHhESC-10-0079; hPSCreg name: WIBRe001-A (RRID:CVCL_9767)] (Lengner et al., 2010), along with autophagy-deficient *ATG5*$^{-/-}$_5 (clones #5) and *ATG5*$^{-/-}$_6 (clone #6) hESC lines (Sun et al., 2023), were cultured feeder-free on Geltrex basement membrane matrix (Gibco, 1413302) in StemFlex Basal Medium (Gibco, A3349401) supplemented with StemFlex 10X Supplement (Gibco, A3349201) and 1 % Penicillin-Streptomycin (Gibco, 15070063) in a humidified incubator with 5 % CO_2 and 5 % O_2 at 37 °C. The hESC lines were passaged every 5–7 days using 0.5 M EDTA (Invitrogen, 15575-038) at 1:5–1:10 ratio. Early passage #18 for *ATG5*$^{+/+}$ hESCs and passage #21 for *ATG5*$^{-/-}$ hESCs were used. The hESCs exhibited normal karyotype (Lengner et al., 2010) that were verified before the study. Expression of pluripotency markers were confirmed in the hESC lines at the start and during the study. The hESCs were frozen down using CryoStor CS10 (StemCell Technologies, 100-1061). For revival, the hESC lines were thawed in StemFlex Basal Medium (Gibco, A3349401) supplemented with StemFlex 10X Supplement (Gibco, A3349201) in the presence of RevitaCell Supplement (Gibco, A2644501). Cell lines were tested negative for mycoplasma and other microbial infections, tested at the start and at quarterly intervals during the study.

Culture of primary human fibroblasts

Primary fibroblasts from a 70-years-old healthy male individual, obtained from the European Collection of Cell Cultures and designated as control 1 (C1) (Hu et al., 2022), were cultured in Advanced DMEM medium, supplemented with 10 % fetal bovine serum, 1 % penicillin/streptomycin and 1 % GlutaMAX (all from Gibco), in a humidified incubator with 5 % CO_2 at 37 °C.

SEAM formation from hESCs

hESCs were seeded onto hESC-qualified Matrigel (Corning) coated plates and cultured in StemFlex medium (Gibco) for 10 days, after which the medium was changed to serum-free differentiation medium (DM) [DMEM with L-Glucose and sodium pyruvate (Sigma-Aldrich) supplemented with 10 % knockout serum replacement (Gibco), 0.1 mM non-essential amino acids (StemCell Technologies), 2 mM L-GlutaMAX (Gibco), 1 % Penicillin-Streptomycin (Gibco), and 55 µM monothioglycerol (Wako)]. After 4 weeks of differentiation, the medium was replaced with epithelial differentiation medium (EDM) [DM together with CnT-PR medium without EGF and FGF2 (CELLnTEC Advanced Cell Systems) at 1:1 ratio containing 20 ng/ml KGF (Qkine) and 10 µM Y-27632 (Tocris) and 1 % Penicillin-Streptomycin (Gibco)] and the cells were cultured for an additional 4 weeks. Non-epithelial cells were removed from the ocular cell lineages by manual pipetting, which was performed around 7 weeks after the initiation of differentiation. The EDM was then replaced with ocular surface epithelial differentiation medium (OSEM) [DMEM/F12 (Gibco) containing 2 % B-27 supplement (Gibco), 20 ng/ml KGF, 10 µM Y-27632 and 1 % Penicillin-Streptomycin)], after which the cells were incubated for an additional 4 weeks to generate the SEAM.

Flow cytometry and cell sorting of SEAM

Cells were dissociated with Accutase (Gibco) and further by pipetting. Dissociated cells were resuspended in ice-cold OSEM and meshed using 40 µm pore size cell strainer (Corning). After washing, samples were stained with SSEA-4 (BioLegend) and ITGB4 (BioLegend) antibodies (see Table S1 for list and dilutions of antibodies) after 10 to 12 weeks of ocular cell lineage differentiation culture using the SEAM method. Small debris was removed by gating in the FSC/BSC(SSC) axis, and the doublets were gated out using the axes of FSC-A/FSC-W and BSC(SSC)-A/BSC(SSC)-W. Double-positive cells were sorted using BD FACSAria Fusion (BD Biosciences) flow cytometer according to the manufacturer's instructions. Sorted cells were collected in OSEM medium for further generation of the 3D LG-like organoids.

Generation of 3D lacrimal gland-like organoids from hESC-derived SEAM

SSEA-4^{+}/ITGB4^{+} sorted cells were cultured in OSEM (see above for composition) on anti-adherence rinsing solution (STEMCELL Technologies) treated round-bottomed 96-well plate for 1 day, at density of 1×10^5 cells per well, for the generation of a spheroid of LG-like progenitors. Subsequently, the spheroids were embedded in 50 % (v/v) of growth factor-reduced Matrigel (Corning) and LG culture medium (LGM) [DMEM/F12 containing 2 % B-27 supplement, 20 ng/ml EGF, 1 % Penicillin-

Streptomycin (all from Gibco) and 10 µM Y-27632 (Tocris)] for approximately 30 days. EVOS FL Cell Imaging System (Thermo Fisher Scientific) was used for bright-field imaging of 3D LG-like organoids.

Immunoblotting analysis
Immunoblotting analysis was performed as previously described (Sun et al., 2023). Cells were lysed in ice-cold RIPA Lysis Buffer [50 mM Tris pH 8, 150 mM NaCl, 0.1 % SDS, 1 mM EDTA, 0.5 % deoxycholate, 1 % IGEPAL (all from Sigma-Aldrich) and Complete Mini Protease Inhibitor Cocktail (Roche)]. The cell lysates were sonicated at 3 × 10 s followed by centrifugation at 12000 × *g* for 30 min at 4 ºC. Protein concentration of the cell lysates was measured by DC Protein Assay (Bio-Rad), and equal amounts of protein (10–40 µg) per sample were subjected to SDS–PAGE and immunoblot analysis. The blots were then incubated in Blocking Buffer [5 % non-fat milk powder (Millipore) in PBS–Tween 20 (Sigma-Aldrich)] for 1 h at room temperature, followed by incubation in primary antibodies (see Table S2 for list and dilutions of primary antibodies) overnight at 4 °C, then washed with PBS (Gibco). The immunoblots were probed with appropriate secondary antibodies (see Table S2 for list and dilutions of secondary antibodies) conjugated to horseradish peroxidase for 1 h at room temperature, then washed with PBS. The chemiluminescent signal was visualised using SuperSignal West Femto Maximum Sensitivity Substrate (Thermo Fisher Scientific) or Amersham ECL Western Blotting Detection Reagent (GE Healthcare) on Amersham Hyperfilm ECL (GE Healthcare) via ECOMAX X-ray Film Processor (PROTEC). Densitometric analysis of immunoblots was performed using ImageJ software (NIH) and data were expressed as a percentage of the control condition that was fixed at 100 %.

Immunofluorescence
Immunofluorescence staining was performed as previously described (Asal et al., 2023; Sun et al., 2023). Cells (hESCs and hESC-derived LG-like organoids) were fixed with 4 % paraformaldehyde (Thermo Fisher Scientific) for 15 min at room temperature, then washed with PBS (Gibco). The LG-like organoids were then incubated with 30 % sucrose (Sigma-Aldrich) solution overnight at 4 °C, embedded in Tissue-Tek O.C.T. (Optimal Cutting Temperature) compound (Sakura), after which they were kept at –80 °C freezer until sectioning, and finally processed as 8 µm thick serial sections in cryostat. The hESCs and the LG-like organoid sections were permeabilized with 0.5 % Triton X-100 (Sigma-Aldrich) for 10 min and incubated with Blocking Buffer [5 % donkey serum (Sigma-Aldrich) in PBS] for 1 h at room temperature. Cells were then incubated with primary antibodies (see Table S3 for list and dilutions of primary antibodies) at 4 °C overnight, then washed in PBS, followed by incubation with appropriate Alexa Fluor conjugated secondary antibodies (See Table S3 for list and dilutions of secondary antibodies) for 1 h at room temperature, then washed again in PBS. The coverslips and the sections were mounted on glass slides with ProLong Gold antifade reagent with DAPI (Invitrogen).

Gene expression analysis
Total RNA extraction from cells was performed using TRIzol reagent (Invitrogen) followed by DNase treatment using the RNase-free DNase set (Qiagen), and the samples were stored at –80 ºC freezer until processed. cDNAs were synthesized using iScript cDNA Synthesis Kit (Bio-Rad). qPCR was performed on the QuantStudio 5 Real-Time PCR instrument (Thermo Fisher Scientific) using SYBR Green Real-Time PCR Master Mix (Applied Biosystem) (for all genes except *SQSTM1* and *ACTB*) or TaqMan Assay (Applied Biosystem) (Hs01061917_g1 for *SQSTM1* and Hs01060665_g1 for *ACTB*) and all reactions were performed in triplicates. Primer sequences are listed in Table S4. Results were analysed using $2^{-\Delta\Delta Ct}$ method (Livak and Schmittgen, 2001) and normalised to the expression of the housekeeping genes *GAPDH* or *ACTB*.

Image acquisition
Immunofluorescence images of fixed cells (hESCs and SEAM) and cryosections (LG-like organoid) were obtained using EVOS FL Cell Imaging System (Thermo Fisher Scientific) with AMG 10x and 20x Plan FL lens, or with LSM880 Confocal Microscope (Zeiss) with 63x/1.2 PlanApo oil immersion lens.

Image analysis of fluorescence intensity and puncta quantification
For immunofluorescence analysis, the fluorescence intensity of cellular markers or quantification of LC3 and p62 puncta were measured relative to the total number of cells analysed using ImageJ (NIH) software. Data were expressed as a percentage of the control condition that was fixed at 100 %. Quantification was performed on ~400–500 cells (for fluorescence intensity measurement) or ~100–300 cells (for measurement of puncta) per sample.

Compound treatment

Compounds used for rescuing the phenotypes of *ATG5*$^{-/-}$ hESC-derived SEAM and LG-like organoids include 1 mM nicotinamide mononucleotide (NMN) (NMN Bio Ltd) and 1 µM melatonin (Tocris), treated for 48 h with replenishment at 24 h. Autophagy modulators used for assessing PAX6 degradation in *ATG5*$^{+/+}$ hESC-derived SEAM were 1 µM rapamycin (Sigma-Aldrich) and 400 nM bafilomycin A_1 (Sigma-Aldrich), treated for 72 h with replenishment every 24 h. Autophagy inhibitors used for analysing hESC-derived SEAM formation were 50 nM bafilomycin A_1 (Sigma-Aldrich) and 1 µM wortmannin (Selleck Chemicals), treated for the last 10 days (with replenishment every 48 h) of the 8-weeks differentiation period.

N-acetyl-β-glucosaminidase (NAG) activity assay

The secretory function of hESC-derived LG-like organoids was assessed by measuring the activity of N-acetyl-β-glucosaminidase (NAG), a lysosomal enzyme in the tear fluid. NAG concentration in supernatants was quantified using NAG Activity Assay Kit (Abcam) following the manufacturer's protocol, as described previously (Asal et al., 2023). The reaction product was detected colorimetrically at 400 nm using a microplate reader (SpectraMax ABS Plus).

Peroxidase activity assay

The secretory function of LG-like clusters in hESC-derived SEAM was assessed by measuring the activity of peroxidase, an enzyme in the tear fluid. Peroxidase activity in supernatants was quantified using Peroxidase Activity Assay Kit (Abcam) following the manufacturer's protocol. The reaction product was detected calorimetrically at 570 nm using Magellan F50 microplate reader (Tecan).

TUNEL assay for apoptotic cells

LG-like organoid cryosections were stained with Click-iT Plus TUNEL Assay for in situ apoptosis detection, Alexa Fluor 488 dye (Invitrogen), according to the manufacturer's protocol and as described previously (Sun et al., 2023). Briefly, cryosections were permeabilised with 0.25 % Triton X-100 (Sigma-Aldrich) for 20 min at room temperature and then washed with deionized water. Cells were incubated at 37 °C for 10 min in TdT reaction buffer, followed by incubation with TdT reaction mixture containing TdT reaction buffer, EdUTP and TdT enzyme for 60 min at 37 °C, washed with 3 % BSA, and finally incubated with Click-iT Plus TUNEL reaction cocktail for 30 min at 37 °C, followed by washes with 3 % BSA. Sections were mounted on glass slides with ProLong Gold antifade reagent with DAPI (Invitrogen). Fluorescence images were acquired with EVOS FL Cell Imaging System (Thermo Fisher Scientific). ImageJ software (NIH) was used to assess the percentage of TUNEL^{+} apoptotic nuclei. The percentage of TUNEL^{+} apoptotic nuclei was calculated from the total number of cells analysed. ~400–500 cells per sample were analysed.

Mitochondrial branch length analysis

Mitochondrial branch length analysis was performed on hESC-derived LG-like organoids cryosections stained with TOM20. Images were preprocessed ImageJ software (NIH) using Unsharp Mask and Enhance Local Contrast (CLAHE) to improve signal-to-noise ratio. The processed images converted to binary and skeletonized. Skeletonized images (~4 images per sample and 200–4000 fragments per image) were analysed using the Analyze Skeleton (2D/3D) plugin to quantify mitochondrial branch length per cell.

ProteoStat assay for aggresomes

Analysis of aggresomes was performed in hESC-derived LG-like organoid cryosections using ProteoStat Aggresome Detection Kit (Enzo), as described previously (Navarro and Ventura, 2014; Sun et al., 2023). Briefly, cryosections were permeabilised with 0.5 % Triton X-100 (Sigma-Aldrich) for 1 h. After washing with DPBS (Gibco), cells were stained with ProteoStat Aggresome Dye (1:2000 in 1 X Assay Buffer) and Hoechst 33342 Nuclear Stain (1:1000 in 1 X Assay Buffer) overnight at 4 °C. After washing, sections were mounted on glass slides with ProLong Gold antifade reagent (Invitrogen) without DAPI. Images were acquired using EVOS fluorescence microscope (Thermo Fisher Scientific) with a Texas Red filter for ProteoStat dye and a DAPI filter for nuclear signal. The percentage of ProteoStat fluorescence intensity was calculated from the total number of cells analysed using ImageJ software (NIH). ~400–500 cells per sample were analysed.

NAD^+ and NADH measurements

NAD^+ and NADH measurements were performed using NAD/NADH Assay Kit (Colorimetric, Abcam), according to manufacturer's instructions and as described previously (Sun et al., 2023). Cells (hESC-derived SEAM) were washed with cold PBS, then homogenized with NAD/NADH extraction buffer and centrifuged 14000 rpm for 5 min at 4 °C. Half of the supernatant was heated to 60 °C for 30 min to decompose the NAD and then immediately cooled in ice. Remaining half of the supernatant was used as NADtotal (NADH plus NAD^+). Both halves of the supernatants were transferred into a 96-well plate and then incubated with Reaction Mix at room temperature for 5 min. NADH Developer was added into each well of 96-well plate and mixed, and the reaction was allowed to cycle at room temperature for 1-2 h. Measurements of optical density (OD) at 450 nm using the EnSpire Multimode plate reader (PerkinElmer) were performed every 20-30 min to detect saturating OD, then normalized to protein concentration via Bio-Rad Protein Assay (Bio-Rad) to measure pmol/µg of NAD^+ and NADH.

Measurement of SIRT1 enzyme activity

Cells (hESC-derived SEAM) were lysed in ice-cold RIPA Lysis Buffer [50 mM Tris pH 8, 150 mM NaCl, 0.1 % SDS, 1 mM EDTA, 0.5 % deoxycholate, 1 % IGEPAL (all from Sigma-Aldrich) and Complete Mini Protease Inhibitor Cocktail (Roche)], following by immunoprecipitation (IP) of SIRT1 with anti-SIRT1 antibody (Proteintech, 6 µg / 50 µL of bead slurry), conjugated to Protein A agarose beads (Invitrogen). For IP reaction, the agarose beads were first washed with lysis buffer and then incubated with 6 µg of primary antibody in lysis buffer for 4 h at 4 ºC. The beads–antibody complex was subsequently washed with lysis buffer and incubated with cell lysate (300 µg of total protein) overnight at 4 ºC. Following centrifugation and washing, the immunoprecipitates were directly subjected to SIRT1 activity measurement using Fluorometric SIRT1 Activity Assay Kit (Abcam), according to manufacturers' instructions. Measurement of fluorescence readout was performed for 30 min with 2 min interval using EnSpire microplate reader, and data were processed with Magellan F50 Software (Tecan). Data were obtained as relative fluorescence units (RFU).

Cell proliferation analysis

Cells (hESCs and hESC-derived SEAM) and LG-like organoid cryosections, immunostained with Ki-67 antibody (cell proliferation marker) and nuclear stained with ProLong Gold antifade reagent with DAPI (Invitrogen), were imaged and assessed for the percentage of Ki-67^+ nuclei in the total number of cells analysed using ImageJ software (NIH). ~100 cells per sample for hESCs, ~200 cells per sample for SEAM, and ~400–500 cells per sample for organoid cryosections were analysed.

Measurement of PAX6 colocalization with LC3 and LAMP1

Analysis of colocalization between PAX6 and LC3, and between PAX6 and LAMP1, in hESC-derived LG-like organoids was performed on confocal microscopy images by the JACoP plugin in ImageJ software (NIH) using Pearson's correlation coefficient. ~100 cells per sample were analysed. 3D reconstruction of PAX6 and LC3 colocalization was generated using Imaris v7.6.5 software (Oxford Instruments).

Proximity ligation assay

hESC-derived LG-like organoid cryosections were stained with Duolink Proximity Ligation Assay (PLA) (Sigma-Aldrich) for in situ protein-protein interaction, according to manufacturer's protocol. Briefly, cryosections were permeabilized with 0.25 % Triton X-100 (Sigma-Aldrich) for 20 min at room temperature, then washed with PBS (Gibco). Blocking was performed using Duolink Blocking Solution for 60 min at 37 °C. After washing with 1 X Wash Buffer A [10 mM Tris pH 7.4, 150 mM NaCl, and 0.05 % Tween-20 (all from Sigma-Aldrich)], sections were incubated with a pair of PLA probes (Sigma-Aldrich) for 1 h at 37 °C. Subsequent washes were followed by incubation with the probe ligation buffer for 30 min at 37 °C, and then with signal amplification buffer for 100 min at 37 °C. Sections were washed with 1 X Wash Buffer B [200 mM Tris pH 7.5 and 100 mM NaCl (all from Sigma-Aldrich)], followed by a final wash in 0.01 X Wash Buffer B. Coverslips and the sections were mounted on glass slides with Duolink In Situ Mounting Media with DAPI (Sigma-Aldrich). Fluorescence images were acquired with LSM880 Confocal Microscope (Zeiss). ImageJ software (NIH) was used to assess the percentage of PLA dots. The percentage of PLA dots were calculated from the total number of cells analysed, ~100 cells per sample.

***In silico* identification of LIR motifs in PAX6**

Identification of putative LC3-interacting region (LIR) motifs within PAX6 was conducted using the iLIR Autophagy Database (https://ilir.warwick.ac.uk) (Jacomin et al., 2016). A BLAST-based search was

performed to compare the PAX6 protein sequence against available protein sequences in the iLIR database, identifying conserved xLIR motifs. Multiple sequence alignments across different species were generated using the ClustalW multiple sequence alignment tool (Thompson et al., 1994) to assess conservation of the identified LIR motifs.

Bulk RNA sequencing

RNA sequencing was performed as previously described (Koçak et al., 2024). The quality and quantity of the total RNA per sample were assessed spectrophotometrically with NanoDrop (Thermo Fisher Scientific). Precise concentration estimation of the samples was done fluorometrically with Qubit (Thermo Fisher Scientific) or Victor Nivo (Perkin Elmer). The integrity of the RNA samples was measured by TapeStation (Agilent Technologies), and the samples with RIN value of 5 or more were considered for the next step. All samples were sequenced on the NovaSeq 6000 Platform PE 2000 cycles (Ilumina). The raw data generated was converted from .bcl to fastQ and demultiplexed by DRAGEN v4.2.7. The transcriptomics data were deposited in the Gene Expression Omnibus (GEO) database, GEO: GSE280811.

Transcriptomics data analysis

For the identification of the genes with a significant change in the expression between *ATG5*$^{+/+}$ and *ATG5*$^{-/-}$_6 hESC-derived SEAM, we used DeSEQ2 package (http://www.bioconductor.org/packages/release/bioc/html/DESeq2.html) (Love et al., 2014). To enhance the accuracy of fold change estimation by reducing background noise, we employed apeglm (Approximate Posterior Estimation for generalized linear model) that uses a heavy-tailed Cauchy prior distribution (https://bioconductor.org/packages/apeglm) (Zhu et al., 2019). We set a threshold for differential expression, considering the Benjamini-Hochberg *P* adj. value < 0.05, |Log2(foldchange)| > 1 as significant. Principal Component Analysis (PCA) and Heatmap of RNA-seq expression Z-scores were plotted using the ggplot2 package (https://ggplot2.tidyverse.org) (Wickham, 2016). Gene ontology (GO) analysis was performed using g:profiler (version e111_eg58_p18_f463989d) with g:SCS multiple testing correction method *P* values < 0.05 (https://biit.cs.ut.ee/gprofiler/gost) (Kolberg et al., 2023). Bubble plots were generated using SRplot (https://www.bioinformatics.com.cn/srplot) (Tang et al., 2023).

Quantification, statistical analysis, and reproducibility

Quantification of data are described under various Methods sections where applicable. Graphical data were expressed as mean ± s.e.m from *n* = ≥3 experimental replicates from 3 independent experiments, and depicted by column graph scatter dot plot (mean ± s.e.m.) using Prism v9.5.0 software (GraphPad). Statistical analyses were performed using Prism v9.5.0 software (GraphPad). Statistical significance (*P* value) on graphical data was determined by unpaired two-tailed Student's t-test with Welch correction or by one-way ANOVA followed by multiple comparisons with two-stage linear step-up procedure of Benjamini, Krieger and Yekutieli. For transcriptomics analysis (*n* = 3 experimental replicates from 3 independent experiments), threshold for differential gene expression was set, considering the Benjamini-Hochberg *P* adj. value < 0.05, |Log2(foldchange)| > 1 as significant. GO: Biological Process terms were selected based on corrected *P* value < 0.05, using g:SCS multiple testing correction method. ****P*<0.001; ***P*<0.01; **P*<0.05; ns (non-significant).

SUPPLEMENTAL REFERENCES

Asal, M., Koçak, G., Sarı, V., Reçber, T., Nemutlu, E., Utine, C.A., and Güven, S. (2023). Development of lacrimal gland organoids from iPSC derived multizonal ocular cells. Front. Cell Dev. Biol. *10*, 1058846. https://doi.org/10.3389/fcell.2022.1058846.

Hu, K., Zatyka, M., Astuti, D., Beer, N., Dias, R.P., Kulkarni, A., Ainsworth, J., Wright, B., Majander, A., Yu-Wai-Man, P., et al. (2022). WFS1 protein expression correlates with clinical progression of optic atrophy in patients with Wolfram syndrome. J. Med. Genet. *59*, 65–74. https://doi.org/10.1136/jmedgenet-2020-107257.

Jacomin, A.-C., Samavedam, S., Promponas, V., and Nezis, I.P. (2016). iLIR database: A web resource for LIR motif-containing proteins in eukaryotes. Autophagy *12*, 1945–1953. https://doi.org/10.1080/15548627.2016.1207016.

Koçak, G., Uyulgan, S., Polatlı, E., Sarı, V., Kahveci, B., Bursali, A., Binokay, L., Reçber, T., Nemutlu, E., Mardinoğlu, A., et al. (2024). Generation of Anterior Segment of the Eye Cells from hiPSCs in Microfluidic Platforms. Adv. Biol. e2400018. https://doi.org/10.1002/adbi.202400018.

Kolberg, L., Raudvere, U., Kuzmin, I., Adler, P., Vilo, J., and Peterson, H. (2023). g:Profiler-interoperable web service for functional enrichment analysis and gene identifier mapping (2023 update). Nucleic Acids Res. *51*, W207–W212. https://doi.org/10.1093/nar/gkad347.

Lengner, C.J., Gimelbrant, A.A., Erwin, J.A., Cheng, A.W., Guenther, M.G., Welstead, G.G., Alagappan, R., Frampton, G.M., Xu, P., Muffat, J., et al. (2010). Derivation of pre-X inactivation human embryonic stem cells under physiological oxygen concentrations. Cell *141*, 872–883. https://doi.org/10.1016/j.cell.2010.04.010.

Livak, K.J., and Schmittgen, T.D. (2001). Analysis of Relative Gene Expression Data Using Real-Time Quantitative PCR and the 2−ΔΔCT Method. Methods *25*, 402–408. https://doi.org/10.1006/meth.2001.1262.

Love, M.I., Huber, W., and Anders, S. (2014). Moderated estimation of fold change and dispersion for RNA-seq data with DESeq2. Genome Biol. *15*, 550. https://doi.org/10.1186/s13059-014-0550-8.

Navarro, S., and Ventura, S. (2014). Fluorescent dye ProteoStat to detect and discriminate intracellular amyloid-like aggregates in Escherichia coli. Biotechnol. J. *9*, 1259–1266. https://doi.org/10.1002/biot.201400291.

Sun, C., Seranova, E., Cohen, M.A., Chipara, M., Roberts, J., Astuti, D., Palhegyi, A.M., Acharjee, A., Sedlackova, L., Kataura, T., et al. (2023). NAD depletion mediates cytotoxicity in human neurons with autophagy deficiency. Cell Rep. *42*, 112372. https://doi.org/10.1016/j.celrep.2023.112372.

Tang, D., Chen, M., Huang, X., Zhang, G., Zeng, L., Zhang, G., Wu, S., and Wang, Y. (2023). SRplot: A free online platform for data visualization and graphing. PloS One *18*, e0294236. https://doi.org/10.1371/journal.pone.0294236.

Thompson, J.D., Higgins, D.G., and Gibson, T.J. (1994). CLUSTAL W: improving the sensitivity of progressive multiple sequence alignment through sequence weighting, position-specific gap penalties and weight matrix choice. Nucleic Acids Res. *22*, 4673–4680. https://doi.org/10.1093/nar/22.22.4673.

Wickham, H. (2016). Data Analysis. In Ggplot2, (Cham: Springer International Publishing), pp. 189–201.

Zhu, A., Ibrahim, J.G., and Love, M.I. (2019). Heavy-tailed prior distributions for sequence count data: removing the noise and preserving large differences. Bioinforma. Oxf. Engl. *35*, 2084–2092. https://doi.org/10.1093/bioinformatics/bty895.
